# Techno-economic optimization for isolated hybrid PV/wind/battery/diesel generator microgrid using improved salp swarm algorithm

**DOI:** 10.1038/s41598-024-52232-y

**Published:** 2024-02-05

**Authors:** Zakaria Belboul, Belgacem Toual, Abderrahman Bensalem, Chaouki Ghenai, Baseem Khan, Salah Kamel

**Affiliations:** 1 Laboratory of Applied Automation and Industrial Diagnostics (LAADI), Faculty of Science and Technology, Ziane Achour University, 17000 Djelfa, Algeria; 2 Renewable Energy Systems Applications Laboratory (LASER), Faculty of Science and Technology, Ziane Achour University, 17000 Djelfa, Algeria; 3 Department of Sustainable and Renewable Energy Engineering, College of Engineering, University of Sharjah, P.O. Box 27272, Sharjah, United Arab Emirates; 4 Renewable Energy and Energy Efficiency Research Group, Sustainable Energy and Power Systems Research Centre, Research Institute for Sciences and Engineering (RISE), University of Sharjah, P.O. Box 27272, Sharjah, United Arab Emirates; 5 Department of Electrical Engineering, Hawassa University, Hawassa, Ethiopia; 6 Department of Electrical Engineering, Faculty of Engineering, Aswan University, Aswan, 81542 Egypt

**Keywords:** Energy infrastructure, Electrical and electronic engineering

## Abstract

The main objective of this study is to develop a new method for solving the techno-economic optimization problem of an isolated microgrid powered by renewable energy sources like solar panels, wind turbines, batteries, and diesel generators while minimizing greenhouse gas emissions. An Improved Salp Swarm Algorithm (ISSA) with a position adaptation mechanism for the salp leader that involves a leader salp that moves about depending on both food availability and its previous position has been proposed to overcome the convergence problem. In the original SSA, as the approach converges, it can no longer find optimal solutions and becomes trapped in a local minimum. Three Microgrid System (MS) configurations are discussed: PV/WT/BESU/DG, PV/BESU/DG, and WT/BESU/DG. The proposed method seeks to find a middle ground between technical criteria and environmental concerns when deciding on PV, WT, BESU, and DG sizes. The findings indicate that the proposed ISSA approach gives superior results compared to other well-known algorithms like the original SSA, the Ant Lion Optimizer (ALO), the Dragonfly Approach (DA), and the Moth-Flame Optimization Algorithm (MFO), which, after significant investigation, has been proven to help determine the appropriate microgrid size. With PV sizes of 10, 9 WT, 24 BESU, and 3 DG, the PV/WT/BESU/DG configuration offers the highest level of cost-effectiveness with Cost of Energy (COE) of 0.2109 $/kWh, Net Present Cost (NPC) of 376,063.8 $, Loss of Power Supply Probability (LPSP) of 4%, Renewable Energy Fraction (REF) of 96%, and CO_2_ emission of 12.4457 tons/year. ISSA is brought up as a possible solution to both the problem of rising energy prices and the difficulties inherent in microgrid design.

## Introduction

Energy conservation and efficiency are prerequisites for the continuous development of civilization. They are essential for maintaining body heat, preparing food, and providing illumination. They are also necessary for modern transportation, communication, healthcare, and industrial systems. Energy availability is essential for a prosperous economy and improving living standards. In addition, energy is a crucial component in the development and transmission of cutting-edge technologies that have the potential to address urgent issues on a worldwide scale, such as poverty, starvation, and damage to the natural environment^1^.

Energy plays a crucial role in our lives, but conventional energy systems have become a cause of concern due to the depletion of fossil fuels, climate change, and global warming. Renewable Energy Sources (RES) have been developed as a sustainable and eco-friendly alternative to traditional systems. Examples of RES include solar, geothermal, wind, hydroelectric, and biomass, which have the potential to provide reliable and sustainable power in the future^2^.

Despite significant advances in RES, rural villages and islands still deal with power shortages. As per the United Nations Development Program (UNDP), over 25% of the global population currently lacks access to electricity. This is especially true for individuals who reside in rural areas. Because of their remote location and the difficulty of developing electrical transmission lines across rugged terrain (such as steep hills or thick jungles), rural populations are frequently unable to afford them. Wind, solar, and hydropower are all examples of renewable resources that may be used to meet the electrical loads in these areas^3^.

The fact that wind and solar energy are not constant sources of power throughout the day is the primary challenge presented by the utilization of such systems for the generation of electricity. Using backup systems like Battery Energy Storage Unit (BESU) and Diesel Generator (DG) is necessary due to the unpredictability of wind and solar power and the inability of power production to adjust to low and extremely high energy demand circumstances^4^. To make up for their intermittent nature, it is essential to implement some energy storage system^5^. This kind of periodic balancing can be done with the help of technologies that store energy, like BESU^6^. DG plays a crucial role as a backup power source to preserve the system's stability, particularly under changing loads and rapidly rising power consumption, as well as when the BESU is low^7^.

Hybrid Renewable Energy System (HRES) combines different techniques of energy production and storage, or it powers a generator with two or more fuel types. HRES are critical in the move away from economies reliant on fossil fuels. HRES has many benefits, such as more renewable energy that can be used immediately and better access to power in rural areas. They also reduce the amount of energy that comes from fossil fuels and use more RES, even those that don't work all the time, which improves energy efficiency and security^8^.

The most practical and affordable alternative for powering off-grid regions is to use renewable energy to create MSs, which improve energy supply^9^. Every MS is made up of distributed energy sources (such as PV, biomass, WT, and fuel cells), distributed energy storage units (such as BESU, supercapacitors, flywheels, and superconducting inductors), and a central control unit. Energy storage technologies are needed to use extra power or compensate for power shortages^10^. Also, static converters like DC/DC, AC/DC, and DC/AC converters help manage energy and voltage adaptation across the various parts of an MS to make it more reliable and cost-effective.

Autonomous microgrids powered by renewable energy are the most practical and cost-effective way to bring electricity to off-grid areas^11^. Considering the technical and economic perspectives, many things make it hard to plan and make the optimal design for such a system. The fact that RES are so weather-dependent makes them unpredictable. In many cases, the size of a microgrid is either too large or too small to provide for the needed load adequately. Excessive system size results in high yearly operating costs and surplus energy production. The opposite is true for undersized MSs, which cannot meet power demand.Microgrids run by renewable energy sources may be advantageous, but this is only the case if their dimensions are optimized. Their energy is controlled efficiently^11^.

Microgrid size has been the focus of discussion in several published studies. Earlier efforts to find the appropriate size can be broken down into three categories^12^. Software tools from the first category, including HOMER Pro, HOMER, HOGA, PVSYST, RAPSIM, and IHOGA, were used to improve the design of MSs^13^. Although easy to use, this category has the disadvantage that users cannot easily choose the best system components. Moreover, users cannot see or access the computations and algorithms^14^. Deterministic approaches, including iterative, analytical, numerical, graphical construction, etc.^15^, are included in the second category. While straightforward, these approaches take a long time to simulate since all system components are scrutinized^15^. The third category is made up of bio-inspired heuristic or metaheuristic algorithms that have been used successfully in many hybrid microgrids to make them more efficient technically and financially^16^.

Size, design, investment, operation, and stability of energy systems, as well as dynamic control, are all examples of complex issues in the energy sector that can be solved with a variety of methods that have been developed through research into artificial intelligence (AI) technologies and that were once thought to be impossible to solve without several simplified assumptions^17^. A branch of AI called “metaheuristic” or “heuristic optimization” uses appropriate computational changes to natural systems to search for global optimal solutions to non-deterministic polynomial time-hard (NP-hard) problems. These issues are incompatible with exact mathematical optimization techniques^18^.

The genetic algorithm (GA) has been used successfully to deal with a hybrid system with many factors^19^, even though it is hard to code. Particle Swarm Optimization (PSO), one of the most popular metaheuristic algorithms, is based on how fish and birds move in groups^20^. Since PSO is so good at solving problems, many different approaches have been tried in different situations^21^. PSO algorithms have been used to study various applications, such as optimizing PV, WT, and BESU^22^. PSO does better than GA because it responds much faster and gets to a solution more quickly^23^.

In other studies, Bukar et al.^11^ used the grasshopper optimization algorithm (GOA) to find the best system configuration in Yobe State, Nigeria, for an MS made up of PV, WT, a BESU, and DG with COE as a fitness function. Similarly, Kaabeche et al.^24^ highlighted the merits of using a firefly algorithm (FA) to identify the best size of an autonomous microgrid. The authors consider COE and the load dissatisfaction rate indices for calculating power supply reliability and system costs, respectively. Fathy et al.^25^ used SSO to find the optimal size of a microgrid with HRES integration. In the Aljouf area of Saudi Arabia, this unit, which is made up of WT, PV, BESU, an inverter, and DG using COE as the objective function, was analyzed using different topologies, such as WT/PV/BESU/DG, WT/BESU/DG, and PV/BESU/DG. Diab et al.^26^ employed four meta-heuristic optimization strategies to discover the best design of a hybrid system with PV, WT, diesel, and BESU. They looked at the four algorithms using the COE and the system's reliability as two ways to compare them. They found that the whale optimization approach gave better results than the other algorithms.

Table 1 provides a comprehensive review of the literature. It includes a summary and a complete analysis of previous work on the design and operation of the microgrid. This table lists information such as the authors of the previous work, the title of the studies, and a brief explanation of their research's conclusions and essential contributions.

**Table 1:** Overview of the employed techniques in enhancing HRES.

Refs.	Year	Location	Microgrid System	Techniques/Tool	Objective Function	Strength	Weakness
^25^	2020	Aljouf, Saudi Arabia	PV/WT/BESU/DG PV/BESU/DG WT/Battery/DG	SSO, GWO, HHO, MVO, WOA and ALO	COE	A clear and detailed research with several results	This study has limited constraints, and the suggested SSO could be explored more
^27^	2020	Yobe State, Nigeria	PV/WT/BESU/DG	PSO, CSA, GOA MOGOA	COE COE/DPSP	Application of single-objective and multi-objective methods (GOA and MOGOA)	The proposed study doesn't analyze different architectures, and it doesn't compare MOGOA to other algorithms
^28^	2021	Dakhla, Morocco	PV/WT/BESU/DG	EO, HHO, GWO, AEFA, and STOA	NPC	Apply a novel meta-heuristic optimization algorithm with many constraints	There weren't many different architectures or configurations analyzed
^29^	2021	Sousse, Tunisia	PV/WT/BESU/DG	RG	TNPC/EC	Real weather data are considered	Algorithm comparison is needed to make sure that the RG algorithm can give the best results
^30^	2022	Yalova, Turkey	PV/WT/BESU/DG	HS, HOMER, ACO and Jaya	ACS	Simple and clear research	The sensitivity analysis is not included
^31^	2023	Sønderborg, Denmark	PV/WT/BESU/DG	MOMFO, NSGA-II, MOPSO, and MOSEO	LCOE/LPSP	The Taguchi method is employed to establish the upper limits of the model's decision variables, while a fuzzy decision-making approach is utilized to acquire the optimal Pareto front	In the proposed scenarios, areas with varying topography can be included in order to improve the accuracy of the decision-maker's analysis, particularly when solar radiation and wind speed are low
^32^	2023	Jiuduansha, China	PV/WT/BESU/DG PV/BESU/DG WT/BESU/DG PV/WT/BESU	HPSODE-FAM, ABC, GA, PSO, and DE	LCOE/LPSP/HDI	A tri-objective function, which incorporates LCOE, LPSP, and HDI, and two strategies for managing energy are implemented	The REF is not considered in the constraints
^33^	2022	Djelfa, Algeria	PV/WT/BESU/DG	MOSSA, MOGOA, MODA, and MOALO	COE/LPSP	Application of a new meta-heuristic optimization algorithm	The MOSSA could be further explored with different configurations of HMS
^34^	2023	Tunisia	PV/WT/BESU/Hydraulic	NSGA-II	LPSP/COST	Multi-objective optimization using an evolutionary algorithm in a remote location	The results obtained are not compared to any other algorithms
^35^	2023	Khartoum, Sudan	PV/WT/BESU/DG	MILP (Gurobi, Python)	NPC/LPSP	Integration Three different solar tracking systems were utilized with a fixed system of PV panels to gain maximum power	The algorithm's application is unclear, and it needs to be compared with other algorithms to get the best results
^36^	2023	Ramnicu Valcea, Romania	PV/WT/BESU/DG PV/WT/BESU/FC PV/WT/BESU	HOMER, iHOGA	COE/NPC	HOMER and iHOGA simulate hybrid system performance. Both programs examine the economic and environmental outlook	The results obtained by applying HOMER and iHOGA are not compared to the meta-heuristic algorithms
^37^	2023	Basra, Iraq	PV/WT/Biomass/BESU/DG	GWCSO, PSO, GA, GWO, CSO, and ALO	ANC/LCOE	Applied a new hybrid meta-heuristic optimization algorithm named GWCSO	This research does not account for reliability, which might decrease the design system's credibility

These algorithms are highly versatile and can avoid the trap of local optima, making them more effective than other approaches. They are based on how living creatures handle problems, offering several advantages that enable them to address any optimization issue. It is worth noting, however, that while they excel at solving particular optimization problems, they may only be successful at solving some of them^11^. Nonetheless, the free lunch hypothesis states that it is always possible to create newer and more effective approaches or algorithms to tackle optimization issues^38^.We opted for ISSA since it is straightforward and needs fewer configuration parameters. It is the optimal approach for fixing the problems above because of how well it has worked when applied to other technical problems.

To date, as far as the authors know, there have been no published studies that specifically applied the ISSA and compared it to other algorithms like the SSA, ALO, DA, and MFO algorithms in the optimal sizing of autonomous microgrids in different configurations such as PV/BESU/DG, WT/BESU/DG, and PV/WT/BESU/DG. The possible innovations and contributions of this paper can be summarized as follows:

- Application of ISSA with a new position adaptation mechanism for salp leaders involves a leader salp that moves based on both food availability and its previous position, addressing the convergence problem in the original SSA.
- The performance and efficiency of the adopted ISSA are validated in comparison to other algorithms, including SSA, ALO, DA, and MFO.
- The novel proposed method aims to identify the optimal size for an MS comprising of PV, WT, BESU, and DG with three different configurations: PV/WT/BESU/DG, PV/BESU/DG, and WT/BESU/DG.

The paper is structured as follows: Models for the various parts of the MS are described in Section "Microgrid system component modeling". The third section covers the weather information and load profiles. In Section "Energy management strategy", we will discuss the Energy Management System. The issues of optimization and a complete design process are covered in Section "Optimization method and strategy". The results of the simulation are discussed in Section "Results and discussion". The paper's conclusion can be found in Section "Conclusion".

## Microgrid system component modeling

**Figure 1:**
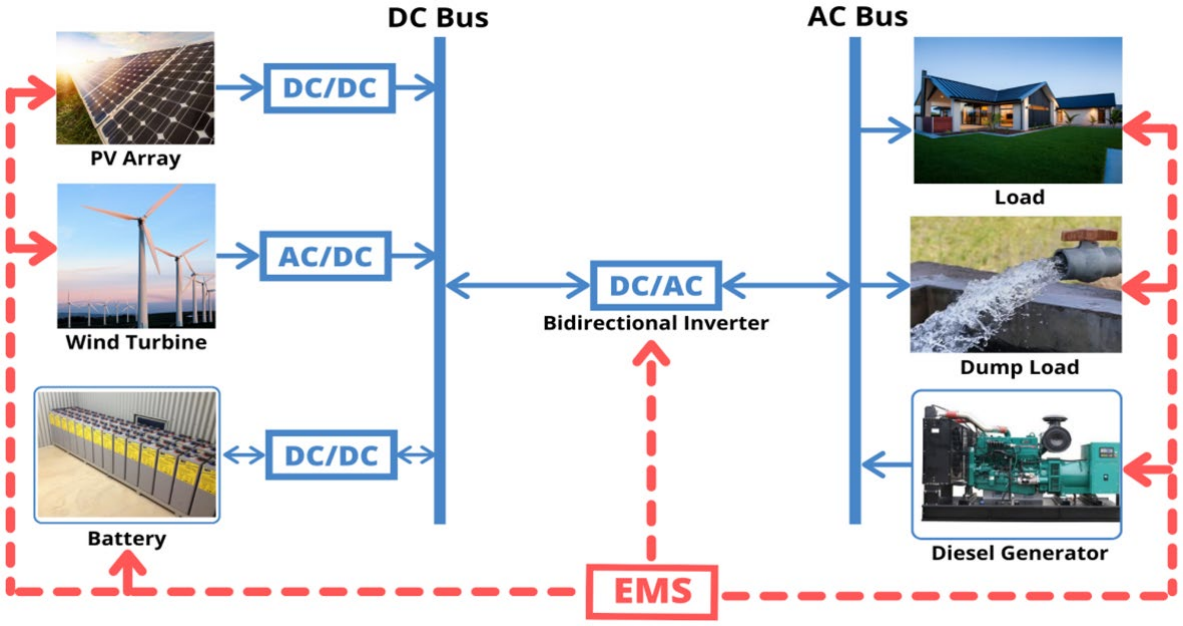
Configuration of the proposed autonomous microgrid system.

MS proposed in this study comprises six main parts: three connected to a DC power bus and the other three to an AC power bus. The DC components of the microgrid system consist of solar PV and WT, along with a battery energy storage unit (BESU). As for the AC components, the demand is met by local load, dump load, and DG acting as a backup power source. An energy management system (EMS) tracks and manages the power-sharing of each component of the MS. The entire layout of the MS is illustrated in Fig. 1. The system is designed to provide energy to a single-phase AC power system with a low voltage of 220 V and a frequency of 50 Hz.

### Solar PV array

There are a number of models available for estimating PV panel output power. However, we utilize a simple model that requires just two inputs: the ambient temperature and the amount of solar radiation^39^. The following equation is used to determine the power generated by the PV panels^39^:

1 $${P}_{p{v}_{-}out}\left(t\right)={P}_{{pv}_{-}ref}\times \frac{{G}_{t}\left(t\right)}{{G}_{{t}_{-}ref}}\times \left[1+{K}_{T}\left({T}_{C}\left(t\right)-{T}_{{C}_{-}ref}\right)\right]$$

where ${P}_{p{v}_{-}out}$ is the power output of PV panels (W), ${P}_{{pv}_{-}ref}$ signifies the rated power (W) of PV panels at the Standard Test Condition STC, which the manufacturer typically specifies, ${G}_{t}$ is the actual solar irradiance of PV panels (kW/m^2^), ${G}_{{t}_{-}ref}$ is the solar irradiance at reference conditions (${G}_{{t}_{-}ref}=1 {\text{kW}}/{{\text{m}}}^{2}$), ${T}_{{C}_{-}ref}$ is the cell temperature at reference conditions (${T}_{{C}_{-}ref}={25}^{^\circ }{\text{C}}$), ${K}_{T}$ is the temperature coefficient of the maximum power, its value is ${K}_{T}=-3.7\times {10}^{-3} (1/^\circ C)$ for the mono and poly-crystalline (Si) solar cells^39^.

The cell temperature ${T}_{C}$ is determined by the following equation^39^:

2 $${T}_{C}(t)={T}_{amb}(t)+\left[0.0256\times {G}_{t}(t)\right]$$

where ${T}_{amb}$ is the ambient temperature.

The overall amount of power produced by a set of PV panels is expressed as follows:

3 $${{P}_{pv}(t)=P}_{p{v}_{-}out}\left(t\right)\times {N}_{pv}\times {\eta }_{pv}$$

where ${N}_{pv}$ is the number of PV panels in the microgrid and ${\eta }_{pv}$ is the efficiency of the PV panels.

### Wind turbine

WT generator has a power output that varies with wind speed, which in turn changes dramatically with altitude at the particular location. The height of the axis utilized for the WT may be calculated from the wind speed recorded by an anemometer. The log law and the power law are employed here to calculate the vertical profile of wind speed at a given location^40^. In this work, the power-law model^40^ is employed to determine the wind speed at the hub height.

4 $${V}_{2}={V}_{1}\times {\left(\frac{h}{{h}_{ref}}\right)}^{\alpha }$$

where ${V}_{2}$ represent the wind speed at the hub height of the WT (m/s), ${V}_{1}$ denote the wind speed at the reference height (m/s), $h$ indicate the hub height of the WT (m), ${h}_{ref}$ represent the reference height of the WT (m). The parameter $\alpha$ represents the friction coefficient.The value of $\alpha$ is influenced by several factors such as topographical characteristics, terrain roughness, wind speed, temperature, height above ground, and time of the year. However, in extreme wind conditions, the friction coefficient should be 0.11 instead of the normal value of 0.20. The commonly accepted value for α is 1/7^40^.

**Figure 2:**
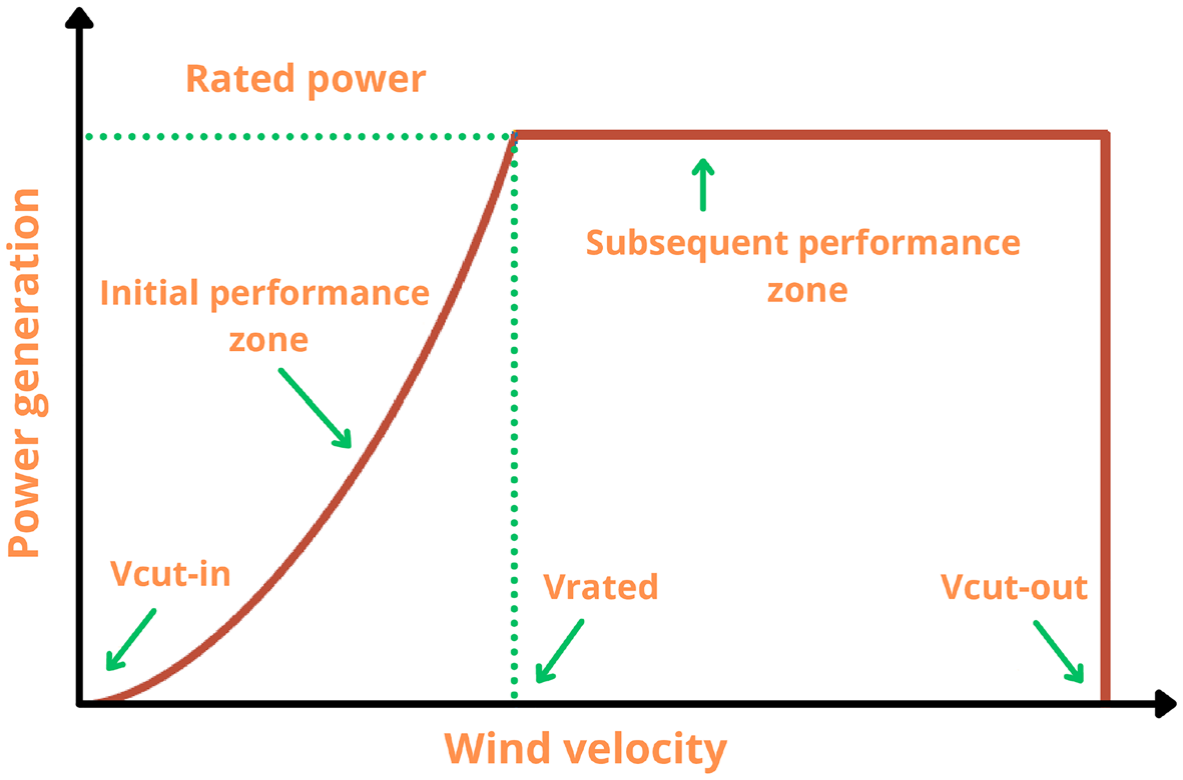
Characteristics curve of WT.

The relationship between wind speed and the power generated by WT is non-linear. The following equation may be used to determine the power generated by each individual WT^40^.

5 $${P}_{wt\_out}(t)=\left\{\begin{array}{ll} 0& V<{V}_{cut-in}\\ { V}^{3}\left(\frac{{P}_{r}}{{V}_{rated}^{3}-{V}_{cut-in}^{3}}\right)-{P}_{r}\left(\frac{{V}_{cut-in}^{3}}{{V}_{rated}^{3}-{V}_{cut-in}^{3}}\right)& {V}_{cut-in}\le V<{V}_{rated}\\ { P}_{r}& {V}_{rated}\le V\le {V}_{cut-out}\\ 0& V>{V}_{cut-out}\\ & \end{array}\right.$$

where ${P}_{r}$ is the rated power of the WT (kW), $V$ is the current time step wind speed (m/s), ${V}_{cut-in}$ is the cut-in wind speed of the WT, ${V}_{rated}$ is the rated wind speed of the WT, ${V}_{cut-out}$ is the cut-out wind speed of the WT. These specifications are provided by the manufacturer of WT. The overall amount of power generated by a set of WT is expressed as follows:

6 $${{P}_{wt}(t)=P}_{{wt}_{-}out}\left(t\right)\times {N}_{wt}\times {\eta }_{wt}$$

where ${N}_{wt}$ is the number ofWT in the microgrid and ${\eta }_{wt}$ is the efficiency of the WT. Figure 2 displays the variation of the output power of the WT generator with wind speed.

### Battery bank

Due to the unpredictability of WT and PV sources, the introduction of BESU into the autonomous microgrid is unavoidable. In this case, HRES uses BESU to store extra energy and use it when renewable energy is not enough or is unavailable. The capacity of the BESU is calculated using the following formula^7^:

7 $${B}_{cap}=\frac{AD\times {E}_{L}}{{\eta }_{inv}{\times \eta }_{Batt}\times DOD}$$

where ${E}_{L}$ represents the load's power demand, $DOD$ denotes the BESU depth of discharge, ${\eta }_{Inv}$ is the inverter's efficiency, ${\eta }_{Batt}$ indicates the BESU efficiency, and the autonomy days ($AD$) are the total number of days that the BESU can meet the load demand without running out.

The required number of BESU that must be connected in parallel, ${N}_{batt\_p}$, is calculated by dividing the total required capacity of BESU, ${B}_{cap}$, by the capacity of a single battery, ${B}_{b}$, as shown below^41^:

8 $${N}_{batt\_p}=\frac{{B}_{cap}}{{B}_{b}}$$

In addition, the number of BESU that must be connected in series, ${N}_{batt\_s}$, is calculated using ${V}_{s}$ and ${V}_{batt}$, the voltages for the DC bus system and the BESU, in volt (V), respectively^41^:

9 $${N}_{batt\_s}=\frac{{V}_{s}}{{V}_{batt}}$$

Finally, the total number of BESU, ${N}_{batt}$, is determined by the product of ${N}_{batt\_p}$ and ${N}_{batt\_s}$, as illustrated below^41^:

10 $${N}_{batt}={N}_{batt\_p}\times {N}_{batt\_s}$$

Accordingly, the total cost of the BESU, ${C}_{C}^{Batt}$, assuming ${C}_{Batt}$ represents the cost of a single battery, will be as follows^41^:

11 $${C}_{C}^{Batt}={N}_{batt}\times {C}_{Batt}$$

Due to the unpredictability of wind speed and intensity of solar irradiance, the autonomy days of the BESU are critical and must be considered when designing the storage system to avoid power shortages generated from RS (PV and WT). When there is an overabundance of energy, it is stored in the BESU and utilized later.

The following equation can be used to indicate the power generated by the BESU^11^:

12 $${P}_{Batt}\left(t\right)=\left({P}_{pv}\left(t\right)+{P}_{wt}\left(t\right)\right)-\frac{{P}_{load}\left(t\right)}{{\eta }_{Inv}}$$

where ${P}_{pv}\left(t\right)$, ${P}_{wt}\left(t\right)$, and ${P}_{load}\left(t\right)$ stand for the generated energy by PV, WT, and load energy demand, respectively, and ${\eta }_{Inv}$ is the efficiency of the inverter.

When ${P}_{Batt}\left(t\right)<0$, it means there is an energy generation deficit. If ${P}_{Batt}\left(t\right)>0$, it implies that energy generation surpasses power demand. In the unusual circumstance where ${P}_{Batt}\left(t\right)=0$, the power provided by RS equals the load power demand.

The state of charge (SOC) of the BESU is a crucial factor that impacts BESU performance and reveals its present capacity when determining the status of the BESU. In this context, the SOC has two modes charging and discharging; when the BESU is in charging mode, if the power produced by RES is higher than the demand, and in discharge mode if the power produced is insufficient to meet the demand. The quantity of charge and discharge at time t is calculated as^11^:

- Charging mode, if; ${P}_{pv}\left(t\right)+{P}_{wt}\left(t\right)>{P}_{load}\left(t\right)$
  13 $${E}_{BT}\left(t\right)={E}_{BT}\left(t-1\right)\times \left(1-\sigma \right)+\left(\left({P}_{pv}\left(t\right)+{P}_{wt}\left(t\right)\right)-\frac{{P}_{load}\left(t\right)}{{\eta }_{Inv}}\right)\times {\eta }_{Batt}$$
- Discharging mode, if; ${P}_{pv}\left(t\right)+{P}_{wt}\left(t\right)<{P}_{load}\left(t\right)$
  14 $${E}_{BT}\left(t\right)={E}_{BT}\left(t-1\right)\times \left(1-\sigma \right)+\left(\frac{{P}_{load}\left(t\right)}{{\eta }_{Inv}}-\left({P}_{pv}\left(t\right)+{P}_{wt}\left(t\right)\right)\right)/{\eta }_{Batt}$$
  where ${E}_{BT}\left(t\right)$ denotes the BESU available capacity at hour $\left(t\right)$ (kWh), ${E}_{BT}\left(t-1\right)$ indicates the BESU available capacity at the hour $\left(t-1\right)$ (kWh), $\sigma$ is the BESU self-discharge rate, ${\eta }_{Batt}$ represents the BESU efficiency (%) during charging and discharging.

Furthermore, the BESU can meet the demand as long as the $SOC\left(t\right)$ is larger than the SOC minimum (${SOC}_{min}$). In the same way, the extra power generated will charge the BESU until $SOC\left(t\right)$ reaches SOC maximum (${SOC}_{max}$), where SOC minimum is 30% and SOC maximum is 100%. The maximum SOC is equal to the total capacity of the BESU (${B}_{batt}$). It is represented as follows^42^:

15 $${B}_{cap}(Ah)=\frac{{N}_{batt}}{{N}_{batt\_s}}\times {B}_{b}(Ah)$$

The maximum allowed depth of discharge (DOD) is expressed as a percentage (%), with 70% considered for this research study. It is impossible to drain the BESU entirely. The DOD value indicates the maximum discharge. The equation below determines the BESU minimum capacity^41^.

16 $${E}_{Batt-min}=\left(1-DOD\right)\times {E}_{Batt-max}$$

Furthermore, the BESU capacity restriction at any hour is represented using Eq. (17)^43^.

17 $${E}_{Batt-min}\le {E}_{Batt}\left(t\right)\le {E}_{Batt-max}$$

where ${E}_{Batt-max}$ denotes the maximum charge quantity of the BESU that is deemed equivalent to the battery's nominal capacity (${B}_{cap}$), ${E}_{Batt-min}$ indicates the minimum charge quantity of the BESU as determined by the maximum depth of discharge ($DOD$), and $DOD$ is the maximum allowable depth of BESU discharge.

### Diesel Generator

In the MS, the DG is used as a backup power source to make up for when the power generated by the renewable resources (PV and WT) and the BESU is inadequate. In order to simplify the DG model's reliance on fuel usage, we use the following equation^7^:

18 $${F}_{DG}(t)=\alpha {P}_{DG}(t)+\beta {P}_{r}$$

where ${F}_{DG}$ is the generator fuel consumption (L/h), $\alpha$ is the fuel curve slope coefficient (L/kWh), $\beta$ is the fuel intercept coefficient (L/kWh), ${P}_{DG}$ is the actual power generated (kW), ${P}_{r}$ denotes the capacity of the generator (kW) or rated power. The values for $\alpha$ and $\beta$ used in this study are $\alpha =0.246$ and $\beta =0.08415$^7^. DG efficiency may be determined as follows^44^:

19 $${\eta }_{overall}={\eta }_{generator}\times {\eta }_{brake-thermal}$$

where ${\eta }_{overall}$, ${\eta }_{generator}$, and ${\eta }_{brake-thermal}$ denote the DG's overall efficiency, generator efficiency, and thermal brake efficiency, respectively. The total power output of a set of DG is defined as follows:

20 $${{P}_{r}=P}_{S\_DG}\times {N}_{DG}$$

where ${P}_{S\_DG}$ is the output power of a single DG and ${N}_{DG}$ is the number of DG in the microgrid.

The cost of fuel ($CF$) throughout the useful lifetime of a power system can generally be expressed as^27^:

21 $$CF={C}_{f}\sum_{t=1}^{8784}{F}_{DG}\left(t\right)$$

where ${C}_{f}$ is the current price of diesel fuel per liter in $US \$/L$.

The carbon dioxide (${CO}_{2}$) emission methodology for DG is predicated on the approach approved by the Intergovernmental Panel on Climate Change (IPCC), which is explained below^45^.

22 $${CO}_{2}={F}_{DG}\times NCV\times EF$$

where ${F}_{DG}$ is the quantity of consumed fuel ($Tons/Year$), $NCV$ denotes the net calorific value of fuel ($TJ/Tons$), and $EF$ refers to the emission factor ($kg {CO}_{2}/TJ fuel$).

The coefficients used for the analysis in this study were obtained from Ref.^45^ and are provided in Table 2.

**Table 2:** Net calorific value coefficient and emission factor.

Fuel utilized	Naphtha
$NCV$	45 $GJ/Tons$
$EF$	73,300 $kg {CO}_{2}/TJ fuel$

### Inverter and converters

Converters are used to convert AC power to DC power and vice versa. Simultaneously, energy flows bidirectionally between the AC and DC buses via the inverter/converter, as illustrated in Fig. 1. Power converters are necessary when the system has both AC and DC components. For example, PV and BESU generate direct current (DC) output while the considered load is AC. The AC to DC/ DC to AC system is modeled based on expected maximum and minimum energy loads and surges, as well as system efficiency. The converter size is determined by peak load demand (${P}_{L}^{peak}$).

The rated power of the inverter, ${P}_{Inv}$, is calculated as follows^39^.

23 $${P}_{Inv}=\frac{{P}_{L}^{peak}}{{\eta }_{Inv}}$$

The efficiency of the inverter can be calculated using the following equation^29^:

24 $${\eta }_{Inv}=\frac{P}{P+{P}_{0}+K{P}^{2}}$$

where $P$, ${P}_{0}$, and $K$ can be determined by the following formula^29^:

25 $$P=\frac{{P}_{out}}{{P}_{n}}, {P}_{0}=1-99{\left(10/{\eta }_{10}-1/{\eta }_{100}-9\right)}^{2}, K=\frac{1}{{\eta }_{100}}-{P}_{0}$$

where ${P}_{n}$ is the rated power of inverter, and ${\eta }_{10}$ and ${\eta }_{100}$ denote the inverter efficiencies at 10% and 100% of its nominal power, respectively, the manufacturer specifies both ${\eta }_{10}$ and ${\eta }_{100}$. The technical and economic parameters of the microgrid components used in this work are listed in “Supplementary file”.

## Meteorological data and load profile

### Location and meteorological condition

**Figure 3:**
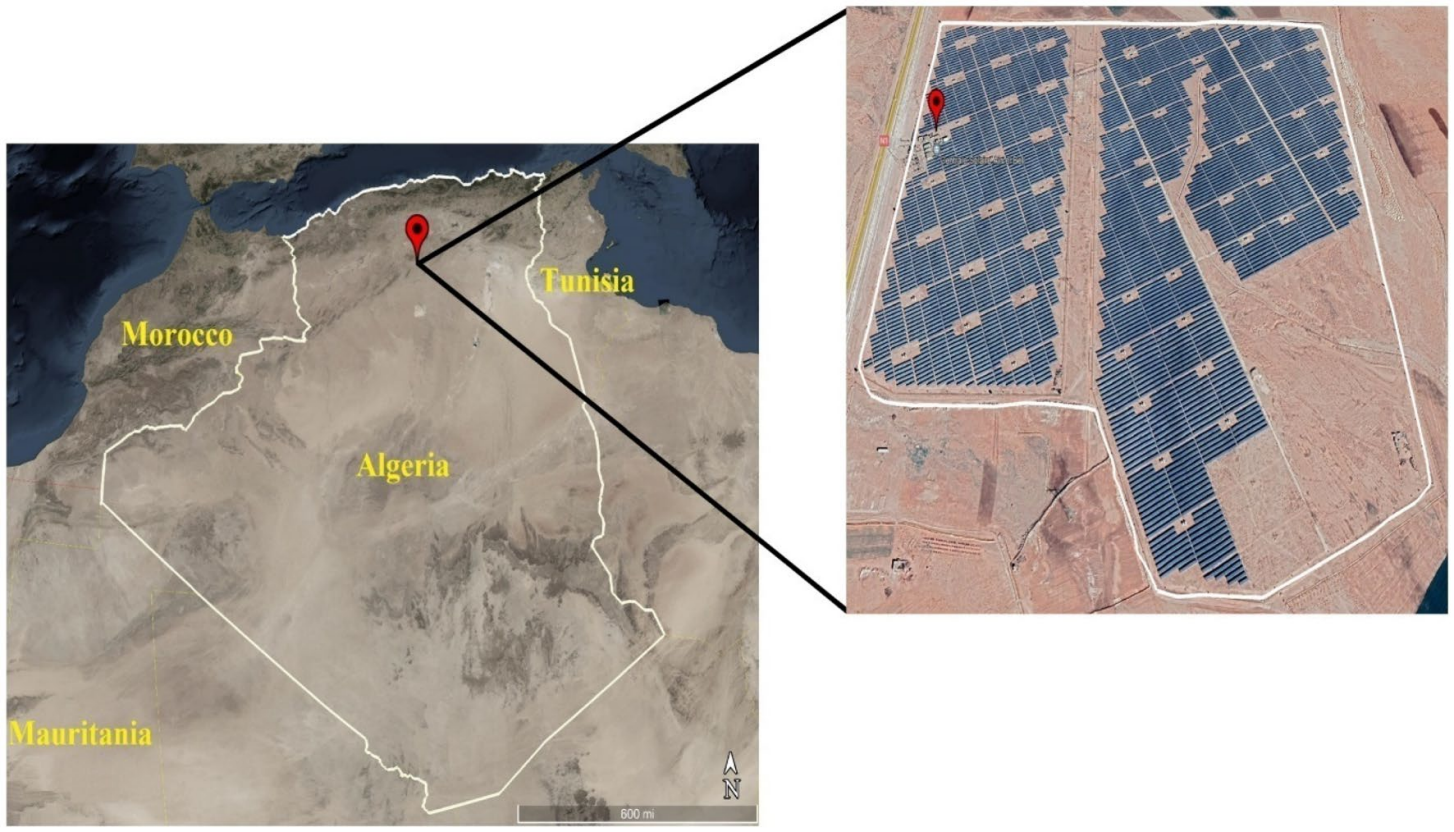
Geographical map of the study location (image courtesy of Google Earth mapping service).

The projected MS, which combines various RES, is intended to be in the town of Aïn El Ibel, located in Algeria's north-central area. The coordinates of this location are 34.346° latitude and 3.163° longitude. The microgrid's proposed construction site is unique because it is located in an area that acts as a bridge between the dry northern high plains and the dry southern desert. The weather conditions in this area are extreme, with extremely sweltering summers and frigid winters. Additionally, the area is characterized by high wind speeds during the winter months. The study site is shown on a map in Fig. 3, and the context for the region and the time frame of data collection are provided in Table 3.

**Table 3:** The geographical coordinates of the studied location.

Specifics	Case Study
Study location Municipality District State Country Longitude Latitude Altitude above sea level	Central PV Aïn El Ibel (SKTM) Aïn El Ibel Aïn El Ibel Djelfa Algeria 3.163° 34.346° 1098 m
Period of measurement	1 January 2020 To 31 December 2020

**Figure 4:**
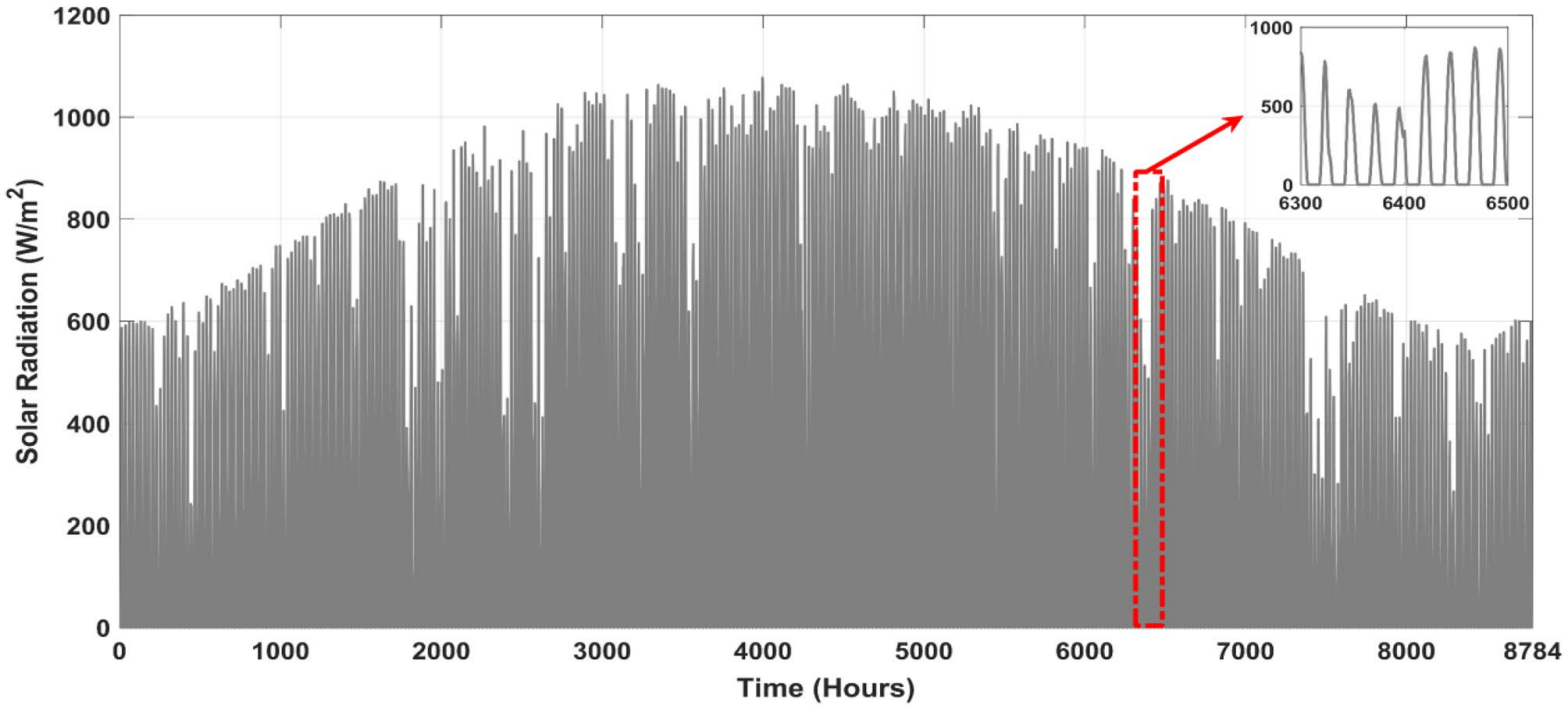
Annual solar radiation in the location under study.

**Figure 5:**
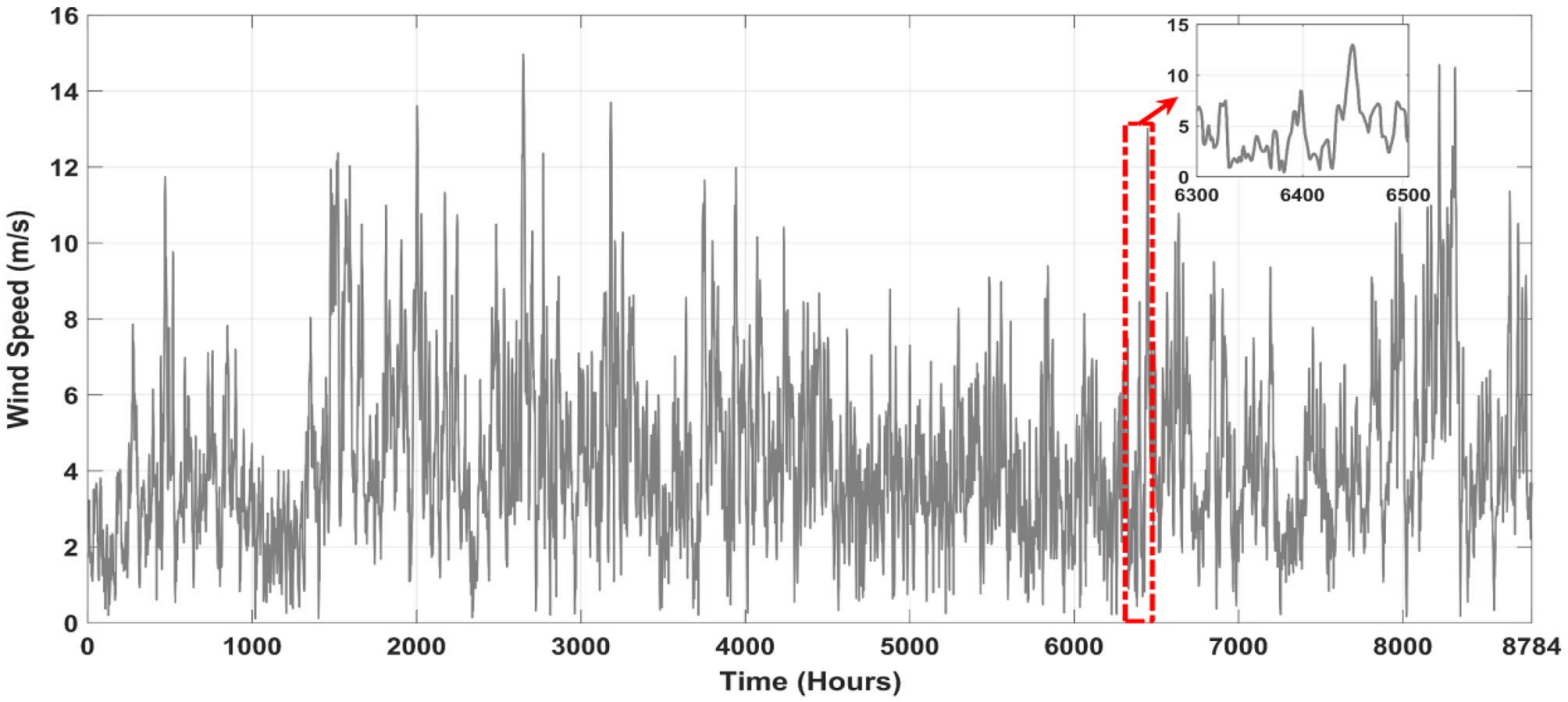
Annual wind speed in the location under study.

**Figure 6:**
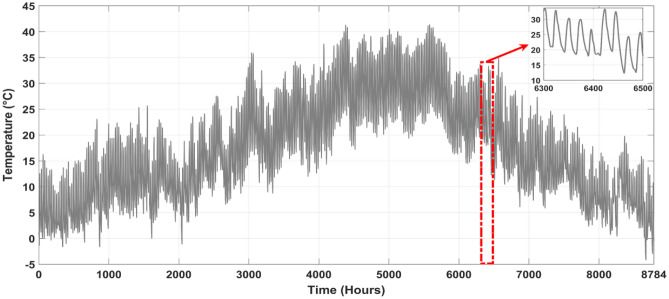
Annual ambient temperature in the location under study.

The main purpose of this study was to simulate wind speed, ambient temperature, and sun irradiance using real-world meteorological data. Our simulations took place in Aïn El Ibel, located at 34.346° latitude and 3.163° longitude. NASA^46^ supplies meteorological data for these models, so you can trust the findings. Solar radiation levels in Aïn El Ibel made it a suitable study location. This is critical for considering the viability of solar power generation. The area's moderate wind speed helps generate wind power. We averaged 0.2357 kW/m^2^ of solar radiation, 17.251 °C of air temperature, and 4.3467 m/s of wind speed throughout the year. The solar radiation profile in Fig. 4 shows a yearly variation. The profile indicated that June, July, and August solar radiation average 0.27 kW/m^2^. Winter solar radiation averages 0.20 kW/m^2^ in December, January, and February. Because the Earth's axis is tilted, in the northern hemisphere, summers are brighter than winters, and vice versa. Figure 5 shows the yearly wind speed variation at 10 m above ground. Winter wind speeds averaged 5.1 m/s across the profile in December, January, and February. In June, July, and August, the wind speed averages 3.8 m/s, the lowest. Many regions see greater winds in the winter when the surface cools. As seen in Fig. 6, ambient temperatures fluctuated throughout the year. The profile shows that June, July, and August average 26.85°C. In December, January, and February, the average temperature drops to 6.85°C. Summer temperatures increase because the sun's rays are unhindered.

### Load assessment

**Figure 7:**
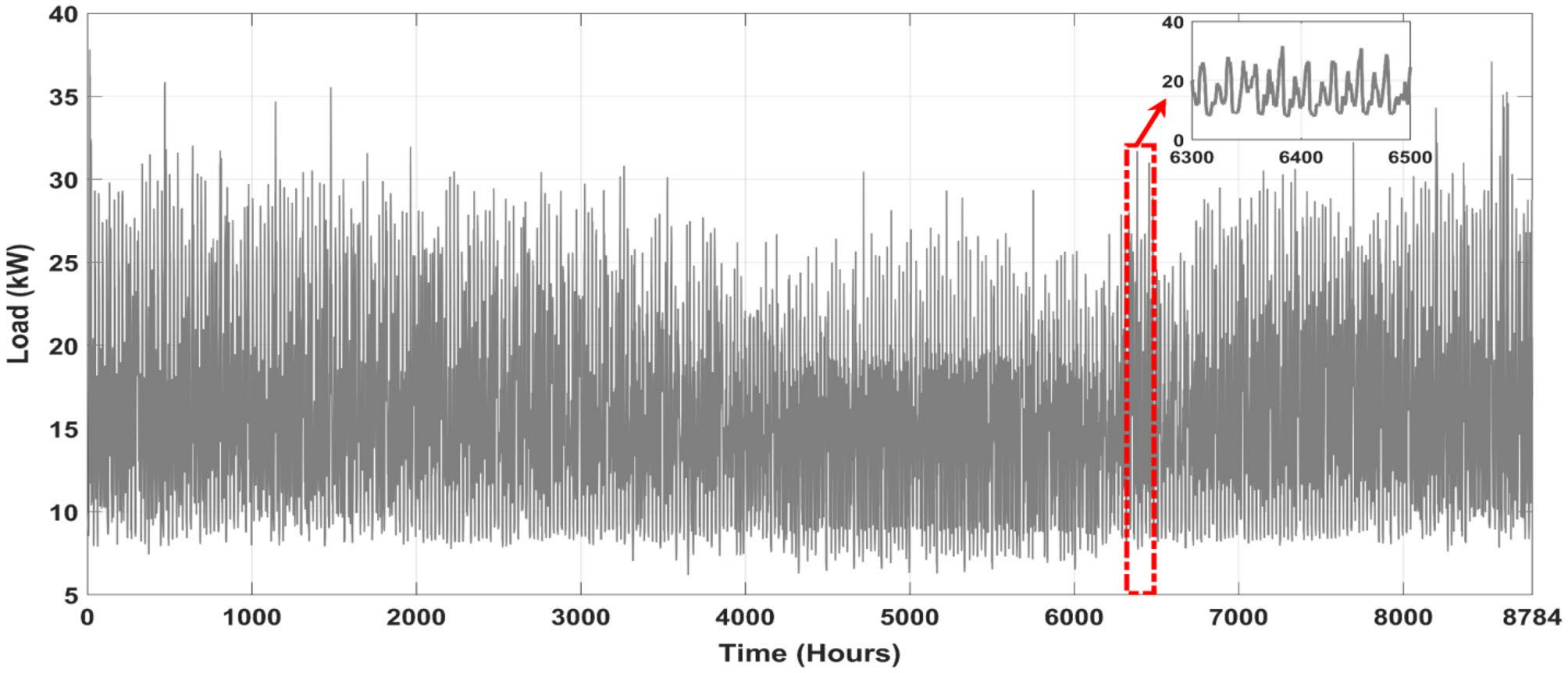
Annual load profile of the location under study.

One of the biggest challenges in microgrid design is load fluctuation. Weather, daily habits, time of day, and human occupancy might create these variances. Load fluctuations may affect system reliability, necessary components, and electrical energy costs. This study focuses on a stand-alone microgrid that supplies energy to a residential area with a cluster of housing units, as illustrated in Table 4. Building a microgrid with RES to suit dwelling units' energy demands is the goal. As shown in Fig. 7, this requires load profile research and an understanding of system component interactions. Microgrid design will incorporate system cost. A reliable, cost-effective system is the goal. RES will dramatically lower microgrid running expenses. Energy storage systems also reduce load variability and improve system reliability.

**Table 4:** Power-consuming devices needed by residential units.

Appliances	Power (W)	Quantity	Electric load (W)
Refrigerator	220	2	440
Television	150	3	450
Mobile Charger	12	6	72
Water Pump	450	2	900
Radio	12	1	12
Lamps Bulb	75	5	375
Lamps CFL	18	8	144
Fluorescent Light	40	5	200
Laptop	46	3	138
Desktop computer	120	2	240
Mixer	450	1	450
Deep freezer	260	1	260
Air conditioner	430	2	860
Washing machine	420	1	420
Microwave	900	1	900

The microgrid of a set of housing units in an off-grid community has been analyzed for a full year, and the findings are shown in Fig. 7 This data is especially useful for analyzing the energy consumption habits of homes and finding opportunities for energy conservation, offering a comprehensive view of power usage throughout the year in order to facilitate comprehension. This data is essential for making educated choices on how to increase energy efficiency and decrease expenditures in the off-grid community.

## Energy management strategy

**Figure 8:**
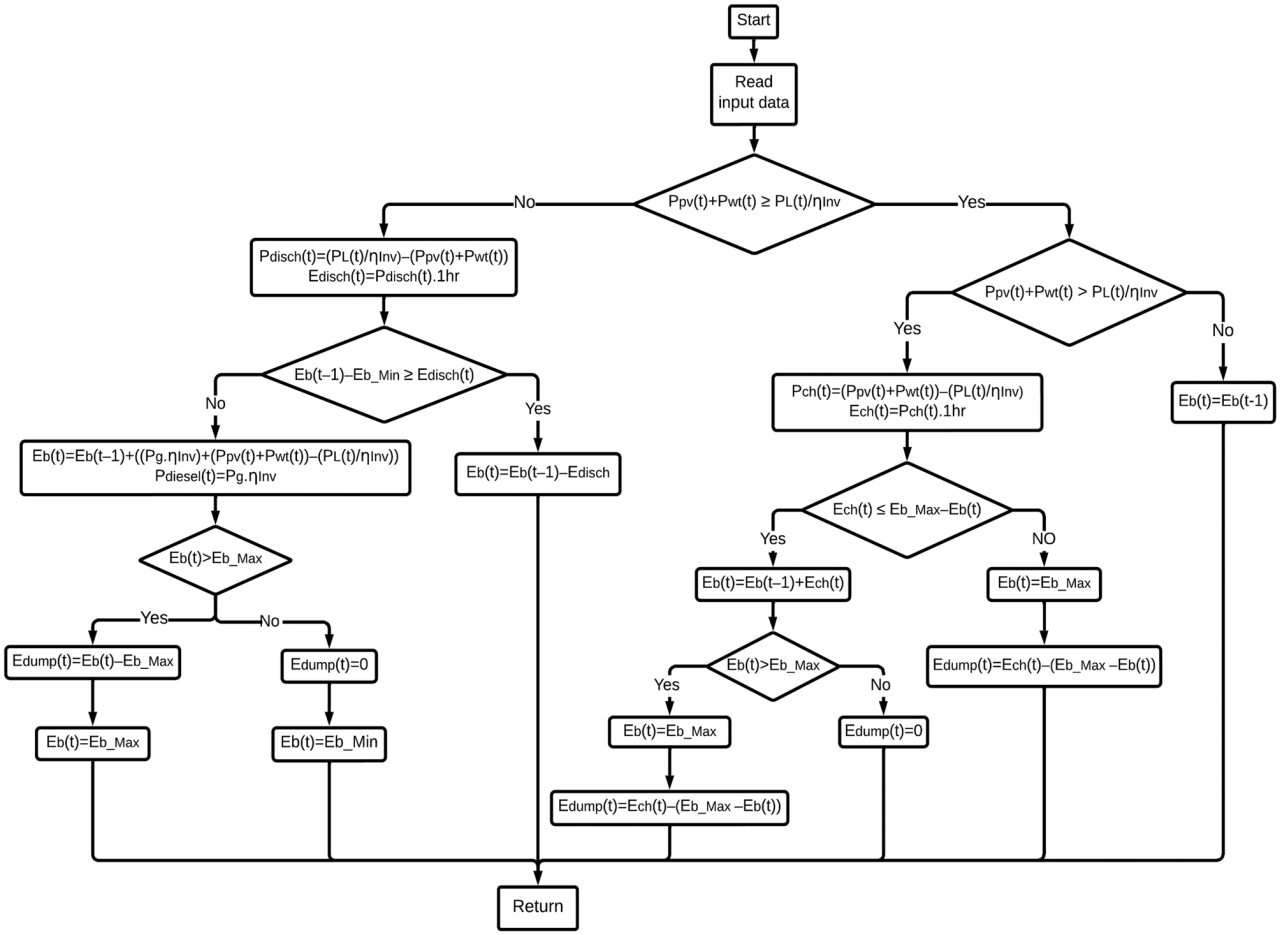
The proposed EMS.

RES' inherent volatility adds complexity to the EMS process. Therefore, it is unrealistic to expect RES alone to meet our energy requirements. In this case, RES, such as PV and WT, may be used in conjunction with the BESU and DG. But it is the system operator's job to optimize renewable energy utilization and minimize the need for the BESU and the diesel engine. Only an EMS that has been well planned and implemented can do this. The purpose of the EMS is to coordinate the distribution of power across the microgrid's multiple components^47^. By decreasing fuel consumption, EMS helps extend the life of BESU and boost the usage of RES. It also helps improve system efficiency, which leads to cost savings and less energy use. Figure 8 displays the flowchart outlining the EMS employed in this study.

The cycle charging-based EMS proposed in this research is implemented using a rule-based method. The algorithm is written mostly using "if" and "then" phrases. The modes of operation are then implemented in a variety of "if" and "then" expressions. The microgrid's energy flow is regulated by several modes, which operate according to a predetermined set of rules. In this study, the EMS is shown in the following modes:

**Mode 1**: The energy generated by RES (such as PV and WT) adequately meets the load energy demand requirements. The surplus energy is put to use to recharge the BESU.

**Mode 2**: When the BESU is fully charged, the energy generated from RES is sufficient to fulfill and surpass the load demand requirements. In this particular instance, the excess energy is used by a "dump load," such as a water pump or a heating system.

**Mode 3**: The energy supplied by renewable resources is insufficient to fulfill the needs of the load. In this instance, the BESU will make up for the shortfall in power production to satisfy the demands of the load.

**Mode 4**: Energy from RS is inadequate to fulfill load demand when the BESU has been exhausted. Here, the DG will be turned on to make up for the difference in energy production so that the load's energy demand is met and to further assure the BESU charge.

## Optimization method and strategy

The optimal sizing for the MS, which consists of PV, WT, BESU, and DG, is determined by an economic-technical strategy. The main aim of optimization is to ensure reliable power supplies at the lowest possible cost. COE is used as an economic criterion, while LPSP is used as a reliability criterion.

### Objective function

COE is the main objective function of MS optimization. Optimization aims to ensure reliable power supplies at the lowest possible cost. The COE is the average of the total NPC ($TNPC$), which includes investment ($Investment$), operation and maintenance (${\text{O}}\&M$), replacement cost ($Replacement$), and fuel cost ($FC$) of the DG. It is common for RES to have minimal operating and maintenance expenses since they do not need fuel, yet capital expenditures are quite expensive.

The COE indicates the cost of energy production per unit. It is the value most often used to estimate the MSs economic profitability, which is outlined as follows^43^.

26 $$COE=\frac{TNPC}{\sum_{h=1}^{8784}{P}_{load}}\times CRF$$

where $TNPC$ is the total NPC, $CRF$ is the capital recovery factor, and ${P}_{load}$ is the hourly loads power consumption, as defined in^43^:

27 $$CRF=\frac{i{\left(1+i\right)}^{n}}{{\left(1+i\right)}^{n}-1}$$

where $n$ is the lifetime of the HRES project (years), which is usually the same as the lifetime of the PV panels, and $i$ is the real interest rate (%). The real interest rate is a function of the nominal interest rate (${i}_{r}$) and the annual inflation rate (${f}_{r}$)^48^.

28 $$i=\frac{{i}_{r}-{f}_{r}}{1+{f}_{r}}$$

The total NPC can be expressed mathematically as follows^27^:

29 $$TNPC=Investment+O\&M+Replacement+FC$$

$Investment$ represents the investment or initial capital cost, which is determined by adding the costs of each component of the MS. $Investment$ is computed using Eq. (30)^29^.

30 $$Investment=\left({N}_{pv}\times {C}_{C}^{PV}\right)+\left({N}_{wt}\times {C}_{C}^{WT}\right)+\left({N}_{batt}\times {C}_{C}^{Batt}\right)+\left({N}_{DG}\times {C}_{C}^{DG}\right)+{C}_{C}^{Inv}$$

where ${N}_{pv}$, ${N}_{wt}$, ${N}_{batt}$, and ${N}_{DG}$ refer to the optimum numbers of PV, WT, BT, and DG, respectively. ${C}_{C}^{PV}$, ${C}_{C}^{WT}$, ${C}_{C}^{Batt}$, ${C}_{C}^{DG}$, and ${C}_{C}^{Inv}$ are the investment costs of the PV, WT, BT, DG, and inverter components, respectively.${\text{O}}\&M$ is the operation and maintenance costs for all system components, which are determined based on the overall cost incurred in O&M over a year. These costs depend on the length of the systems life and the interest rate. It is expressed as follows^29^:

31 $${\text{O}}\&M=\left(\left({N}_{pv}\times {C}_{O\&M}^{PV}\right)+\left({{N}_{wt}\times C}_{O\&M}^{WT}\right)+\left({N}_{batt}{\times C}_{O\&M}^{Batt}\right)+\left({N}_{DG}{\times C}_{O\&M}^{DG}\right)\right)\times \left(\frac{(1+i{)}^{n}-1}{i(1+i{)}^{n}}\right)$$

where ${C}_{O\&M}^{PV}$, ${C}_{O\&M}^{WT}$, ${C}_{O\&M}^{Batt}$, and ${C}_{O\&M}^{DG}$ represent the O&M costs for PV, WT, BT, and DG, respectively. The O&M costs of the inverter and converters are overlooked. The replacement cost of the MS components can be determined as follows^29^:

32 $$Replacement={C}_{Replacement}^{Batt}+{C}_{Replacement}^{DG}+{C}_{Replacement}^{Inv}$$

33 $${C}_{Replacement}^{Batt}={N}_{batt}\times {C}_{R}^{Batt}\times {\sum }_{j=1}^{\left(\frac{n}{{n}_{Batt}} -1\right)}\left(1+\frac{1}{(1+i{)}^{j{n}_{Batt}}}\right)$$

34 $${C}_{Replacement}^{DG}={{N}_{DG}\times C}_{R}^{DG}\times {\sum }_{j=1}^{\left(\frac{n}{{n}_{DG}} -1\right)}\left(1+\frac{1}{(1+i{)}^{j{n}_{DG}}}\right)$$

35 $${C}_{Replacement}^{Inv}={C}_{R}^{Inv}\times {\sum }_{j=1}^{\left(\frac{n}{{n}_{Inv}} -1\right)}\left(1+\frac{1}{(1+i{)}^{j{n}_{Inv}}}\right)$$

where ${C}_{R}^{Batt}$, ${C}_{R}^{DG}$, and ${C}_{R}^{Inv}$ represent the replacement costs of the BESU, DG, and inverter, respectively. As both the PV panels and the WT have a 25-year lifetime, their replacement is not considered.${n}_{Batt}$, ${n}_{DG}$, and ${n}_{Inv}$ are the lifetimes of the BESU, DG, and inverter, respectively.

The expression for the fuel cost ($FC$) has been detailed in the equation below.

36 $$FC=\frac{CF}{CRF}$$

### Constraints

During the optimization process, several constraints and limitations are considered to avoid undesirable outcomes because of the microgrid's operational and physical restrictions. The following constraints are considered:

#### Loss of power supply probability

One of the most important indicators for evaluating the longevity of a hybrid MSs capacity and performance is its reliability. In this research, LPSP is used to measure the dependability of a system. LPSP is a design factor that may take on values between 0 and 1. If it has a value of 0, all of the loads electrical needs have been fulfilled. However, if LPSP is 1, the load power demand cannot be supplied. Below is the equation used to determine the LPSP^49^:

37 $$LPSP=\frac{\sum_{t=1}^{8784}LPS\left(t\right)}{{\sum }_{t=1}^{8784}{P}_{load}\left(t\right)}$$

The Loss of Power Supply (LPS) at every given instant “t” may be determined using Eq. (38)^49^.

38 $$LPS\left(t\right)={P}_{load}\left(t\right)-\left({P}_{pv}\left(t\right)+{P}_{wt}\left(t\right)+{P}_{Batt}\left(t\right)+{P}_{DG}\left(t\right)\right)$$

where ${P}_{pv}\left(t\right)$, ${P}_{wt}\left(t\right)$, ${P}_{Batt}\left(t\right)$, and ${P}_{DG}\left(t\right)$ are the power outputs of the PV panels, WT, BESU, and DG, respectively.

In this analysis, the worst-case scenarios are used to assess the systems reliability, as follows^38^:

39 $${P\left(t\right)}_{load}>{P\left(t\right)}_{generate}$$

where ${P\left(t\right)}_{generate}$ is the amount of power produced. In this case, it's vital to remember that the total energy demand of the load is greater than the sum of the energy output from all sources.

#### Renewable energy fraction

The pursuit of the energy transition from conventional energy production to a renewable energy project is not a straightforward process. The objective here is to increase the use of renewable energy. REF defines the proportion of energy produced from renewable sources relative to non-renewable sources (DG) within the microgrid and is represented as follows^38^:

40 $$REF=\left(1-\frac{\sum {P}_{DG}}{\sum {P}_{pv}+\sum {P}_{wt}}\right)\times 100$$

When the REF reaches 100%, the system is in optimal condition and depends only on electricity provided by RES. When it equals 0%, it indicates that the DG is making the same amount of energy as RES.

In addition to the aforementioned constraints, the following constraints must also be adhered to:

41 $$\left\{\begin{array}{c}0\le {N}_{pv}\le {N}_{pv}^{max}\\ 0\le {N}_{wt}\le {N}_{wt}^{max}\\ 0\le {N}_{batt}\le {N}_{batt}^{max}\\ 0\le {N}_{DG}\le {N}_{DG}^{max}\\ LPSP\le {LPSP}^{max}\\ {REF}^{min}\le REF\\ {AD}^{min}\le AD\end{array}\right.$$

### Design variables

The number of PV (${N}_{pv}$), the number of WT (${N}_{wt}$), the number of BESU (${N}_{batt}$), and the number of DG (${N}_{DG}$) are the design variables taken into account in this research. The following are the lower and upper limits on the suggested design variables:

42 $$\text{Design variables }=\left\{\begin{array}{c}10\le {N}_{pv}\le 45\\ 5\le {N}_{wt}\le 25\\ 1\le {N}_{batt}\le 24\\ 1\le {N}_{DG}\le 5\end{array}\right.$$

### Algorithm

**Figure 9:**
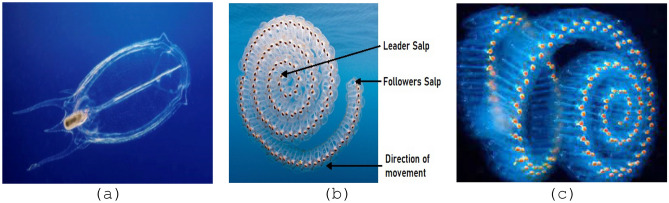
In the realm of a salp swarm, we observe three shapes: (**a**) an individual salp, (**b**) a chain of salps, (**c**) a collective swarm of salps.

SSA is a recently developed metaheuristic optimization approach, first proposed by Mirjalili et al.^50^. In their work, the authors presented a theoretical framework that describes the foraging behavior of salps and highlights their remarkable cognitive abilities. This research was conducted in 2017. Salps have a behavioral response whereby they aggregate into chains when encountering a food supply, facilitating their foraging efficiency. According to the illustration shown in Fig. 9b, the salp colonies are seen to be guided by a dominant salp. This particular salp assumes the responsibility of overseeing and organizing the collective efforts of the other salps in their pursuit of capturing the food supply. Figure 9a illustrates the morphological characteristics of salp organisms, which often aggregate to form salp chains. Figure 9c illustrates the sequence of salps in a visual representation. There is a notable resemblance between the tissues of salps and jellyfish. The movement requirements of both entities demonstrate a significant level of resemblance, as shown in citation^50^.

#### Single-objective SSA

The mathematical model developed by the SSA accurately simulates the hunting behavior seen in swarms of salps, a species belonging to the Salpidae family. The model divides the population of the salp swarm into two distinct types, namely leaders and followers. In each iteration, instead of individually searching for the ideal value, every non-leader salp mimics the movement of the previous salps.

The leader salp, ${x}_{j}^{1}$, advances towards the food supply (${F}_{j}$) located in the search area, while the subordinate salps can travel towards the remaining salps. The salps are defined in a search space with n dimensions. This corresponds to the number of factors specific to the problem being addressed. Consequently, a two-dimensional matrix named ${X}_{i}$, with dimensions $N\times d$, is employed to store the positions of all the salps, as stated in Equation^50^:

43 $${X}_{i}=\left[\begin{array}{llll}{x}_{1}^{1}& {x}_{2}^{1}& \dots & {x}_{d}^{1}\\ {x}_{1}^{2}& {x}_{2}^{2}& \dots & {x}_{d}^{2}\\ \vdots & \vdots & \dots & \vdots \\ {x}_{1}^{N}& {x}_{2}^{N}& \dots & {x}_{d}^{N}\end{array}\right]$$

The leader position is updated in accordance with the following equation^50^:

44 $${x}_{j}^{1}=\left\{\begin{array}{ll}{F}_{j}+{c}_{1}\left(\left(u{b}_{j}-l{b}_{j}\right){c}_{2}+l{b}_{j}\right)& {c}_{3}\ge 0\\ {F}_{j}-{c}_{1}\left(\left(u{b}_{j}-l{b}_{j}\right){c}_{2}+l{b}_{j}\right)& {c}_{3}<0\end{array}\right.$$

The position vector, denoted as ${x}_{j}^{1}$, represents the location of the first salp, which acts as the leader. Similarly, the position vector ${F}_{j}$ corresponds to the feeding supply position in the ${j}^{th}$ dimension. The upper and lower bounds of the ${j}^{th}$ dimension is indicated by $u{b}_{j}$ and $l{b}_{j}$, respectively. In this context, ${c}_{1}$, and ${c}_{2}$ are random numbers within the range of [0,1]. As per Eq. (44), the leader only adjusts its position based on the food source, disregarding other factors.

SSA coefficient ${c}_{1}$ is one of the most crucial factors because it strikes an equilibrium between investigation and utilization. It is described as follows^51^:

45 $${c}_{1}=2{e}^{-{\left(\frac{4l}{L}\right)}^{2}}$$

where, $L$ signifies the maximum number of iterations, while $l$ represents the current iteration.

The followers adjust their positions according to the following equation^50^:

46 $${x}_{j}^{i}=\frac{1}{2}a{t}^{2}+{v}_{0}t$$

where $i\ge 2$, ${x}_{j}^{i}$ represents the position vector of the ${i}^{th}$ follower salp in the ${j}^{th}$ dimension. Furthermore, $t$ denotes time, ${v}_{0}$ corresponds to the initial speed, and a represents the ratio of $a=\frac{{v}_{final}}{{v}_{0}}$, where $v=\frac{x-{x}_{0}}{t}$.

The difference between iterations is equivalent to 1 signifying the repetition based on a specific sampling unit in optimization time, and by taking into account that ${v}_{0}=0$, the aforementioned equation can be stated as follows^50^:

47 $${x}_{j}^{i}=\frac{1}{2}\left({x}_{j}^{i}+{x}_{j}^{i-1}\right)$$

where $i \ge 2$ and ${x}_{j}^{i}$ represents the locations vector of the follower Salps in the ${j}^{th}$ dimension, the collective behavior of the salp chains can be comprehended and mathematically reproduced based on the simulations outlined in the aforementioned formulas.

**Figure 10:**
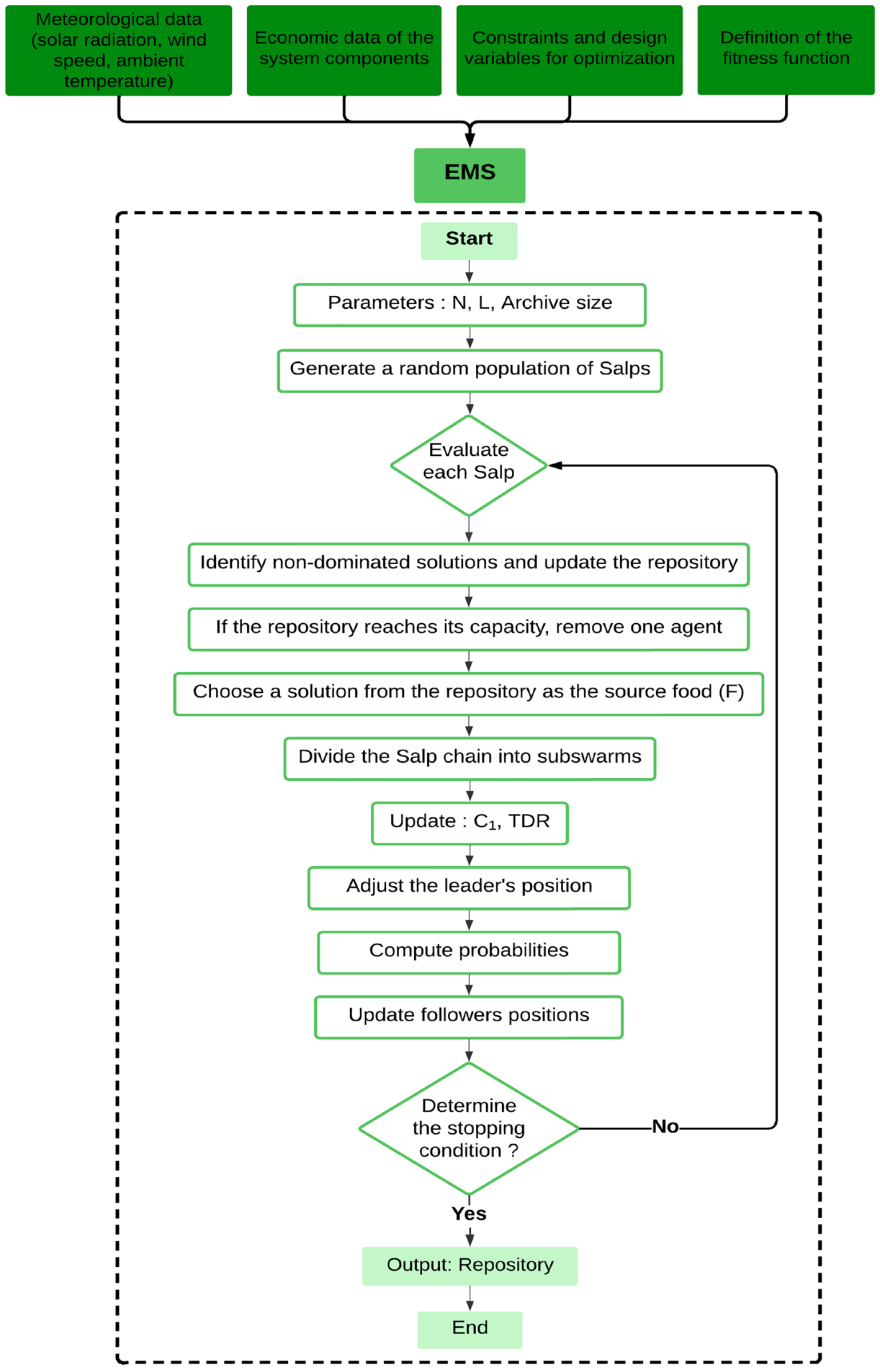
Flowchart of SSA method.

To solve the sizing problem, SSA is used to follow several procedures. The proposed procedure of action is defined in detail in Fig. 10

#### Improved SSA

The equation denoted as (48) delineates the mechanism by which salps are reintroduced into the search region subsequent to their departure or in instances when specific salps have strayed outside its boundaries.

48 $${x}_{j}^{i}=\left\{\begin{array}{c}l{b}_{j} if {x}_{j}^{i}\le l{b}_{j}\\ u{b}_{j} if {x}_{j}^{i}\ge u{b}_{j}\\ {x}_{j}^{i} otherwise\end{array}\right.$$

SSA may not be suitable for more complex problems due to its tendency to become stuck in local optima, despite its ability to provide satisfactory outcomes when compared to other well-recognized algorithms. Based on the equation denoted as (44), the displacement of the dominant salp within the SSA is contingent upon the comparative nutritional value of the accessible food sources and the relative location of the most superior salp within the whole population. This finding implies that in each iteration, the SSA algorithm functions by maintaining the leader salp in close proximity to a fixed point while the other salps (following) change their locations dynamically to converge with the leader. Once the iterative process reaches convergence, it loses the ability to explore globally optimum solutions and becomes confined to a local minimum. To effectively tackle this matter and boost the search capabilities and flexibility of the SSA, a refined iteration of the algorithm, referred to as the ISSA, is presented. The suggested ISSA model incorporates a leader salp that exhibits movement patterns influenced by the availability of food resources and its prior spatial location. This is undertaken in order to enhance the efficacy and investigative capacities of the technique. The determination of the leading salps' position update is accomplished by the use of the following equation^52^:

49 $${x}_{j}^{1}=\left\{\begin{array}{ll}{x}_{j}^{i}+{c}_{1}\left({F}_{j}-{x}_{j}^{i}\right)& {c}_{3}\ge 0.5\\ {x}_{j}^{i}-{c}_{1}\left({F}_{j}-{x}_{j}^{i}\right)& {c}_{3}<0.5\end{array}\right.$$

In Eq. (49), the variable ${c}_{1}$ signifies a time-varying value derived from Eq. (45), while ${c}_{3}$ denotes a random number ranging from 0 to 1. By adopting this approach, the SSA algorithm facilitates both exploration and a more efficient global search across the entire search area. In order to enhance the efficacy of the proposed ISSA, the followers modify their locations based on the subsequent equation^52^:

50 $${x}_{j}^{i}={c}_{1}\times rand\left({x}_{j}^{i}+{x}_{j}^{i-1}\right)$$

In Eq. (50), instead of the conventional fixed value of 0.5, a random time-varying element is employed. This element enhances the global search capability of the algorithm in its early iterations and fosters local search in later iterations. Moreover, within the proposed ISSA framework, upon the creation of each algorithm, the salp with the highest fitness value is substituted for a randomly generated salp.

**Figure 11:**
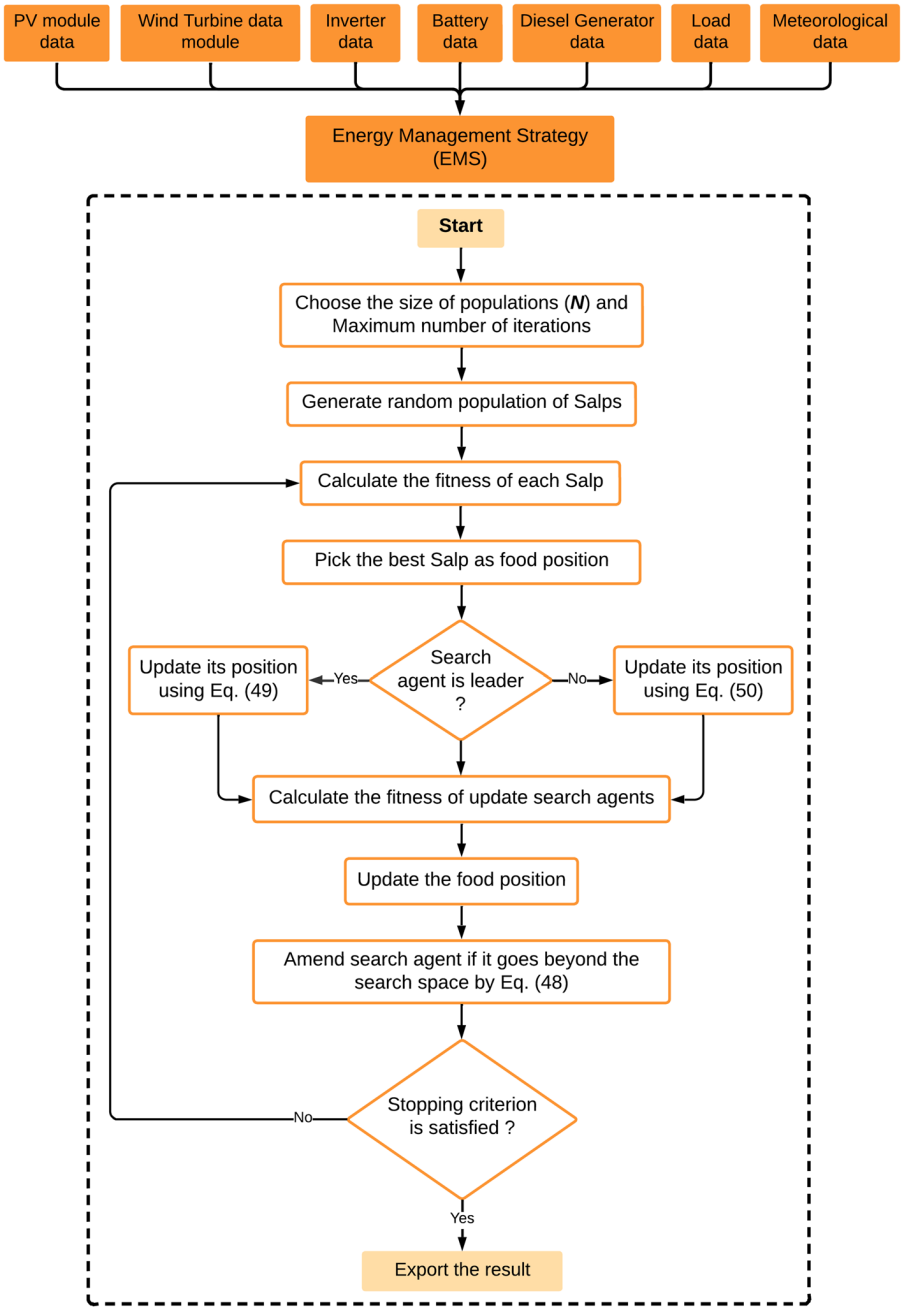
Flowchart of the proposed ISSA method.

In order to address the issue of sizing, ISSA employs a number of procedures. Figure 11 provides a very detailed explanation of the suggested strategy. Upon initiation of the simulation, the ISSA algorithm randomly distributes particles within the search landscape, as is typical of optimization algorithms, with the user-defined constraints dictating the bounds of the search. As per the governing equations of the algorithm, the particles navigate through the search landscape with the aim of optimizing the objective function that has been defined.

The authors have done a number of tests to see how well the ISSA works. In these tests, the ISSA's performance is compared to that of other optimization methods in various scenarios. For example, when solving optimization problems in the coordinated design of damping controllers for a power system with a UPFC, the ISSA was found to be much better in terms of stability, accuracy, and flexibility. The proposed method also works well to stop oscillations in a power system with more than one machine when there are faults^52^. By changing the hyperparameters of a backpropagation neural network that uses chaotic mapping and a decay factor, ISSA was also used to improve the prediction of tool wear. The algorithm is tested on benchmark functions, showing better solution accuracy and stability^53^. In another study^54^, ISSA is applied to enhance the performance of a blind digital modulation detection technique. The improvements to SSA include introducing an inertia weight, dynamically varying the weight factor in the position update formula and employing an opposition-based learning technique to overcome premature convergence and evolution stagnation. ISSA is proposed for feature selection in high-dimensional medical data classification and compares the performance of this algorithm in terms of classification accuracy and execution time, where ISSA incorporates an inertia weight parameter to enhance exploration and exploitation capabilities. The algorithm is tested on 23 UCI datasets. Results show that ISSA outperforms other optimization algorithms in terms of classification accuracy and feature reduction^55^.

## Results and discussion

This research paper discusses the optimal size of an autonomous microgrid that utilizes HRES comprises of PV, WT, and BESU. It also uses the DG system as a backup source. Figure 1 shows the proposed microgrid. The system meets the energy demand of a group of houses for a residential unit. Furthermore, the paper presents analyses of the total NPC, the energy generation contribution of the various elements, CO_2_ emissions, and the energy flow of the designed microgrid. This is based on the case study results. By examining the costs incurred and benefits accrued from the system and its technical feasibility, the proposed microgrid is evaluated for its technical feasibility and economic viability. The results of the analyses provide insights into the economic and financial aspects and technological aspects of the proposed microgrid, determining its practicality and long-term viability. The analyses below are made for three different MS configurations:

- PV/WT/BESU/DG;
- PV/BESU/DG;
- WT/BESU/DG.

The PV/WT/BESU/DG configuration combination uses four distinct energy generation sources. This combination utilizes two RES, namely PV and WT, as well as the BESU and DG, to generate electricity. The combination of the PV/BESU/DG configuration combines three diverse sources of energy generation, including one renewable source, PV, plus the BESU and DG. Finally, the WT/BESU/DG configuration combination also utilizes one renewable source, the WT, plus the BESU and DG to generate electricity.

MATLAB R2018a provided a platform for system's implementation. The Windows 10 Pro Version 21H2 (64-bit) PC with a 1.80 GHz Intel Core i7-10510U CPU and 16 GB of RAM ran tests efficiently.

### Validation of ISSA algorithm

This research involves using five optimization techniques to solve the optimization design problem highlighted in this paper. The five algorithms are the ISSA, SSA, ALO, DA, and MFO. In order to analyze the efficiency of the five suggested approaches, ISSA, SSA, ALO, DA, and MFO, they were executed over the course of 100 iterations. To ensure accuracy and consistency, each approach was subject to specific control parameters carefully selected for future reference. These control parameters were unique to each approach, as they were tailored to suit the specific characteristics and requirements of each method. The control parameters were documented in Table 5, which provides a detailed overview of the various control parameters utilized in each approach.

**Table 5:** Optimization control parameters for each algorithm.

Techniques	Parameters
ISSA	Population size: 10 Independent simulation run: 30 The coefficient ${c}_{1}$: Eq. (45) The coefficient ${c}_{2}$: rand
SSA	Population size: 10 Independent simulation run: 30 The coefficient ${c}_{1}$: Eq. (45) The coefficient ${c}_{2}$: rand The coefficient ${c}_{3}$: rand
ALO	Population size: 10 Independent simulation run: 30 ${c}^{t}=\frac{{c}^{t}}{I}$,${d}^{t}=\frac{{d}^{t}}{I}$
DA	Population size: 10 Independent simulation run: 30 $w=0.9-0.2$, $s=0.1$ $a=0.1$, $c=0.7$, $f=1$,$e=1$
MFO	Population size: 10 Independent simulation run: 30 $b=1$

**Figure 12:**
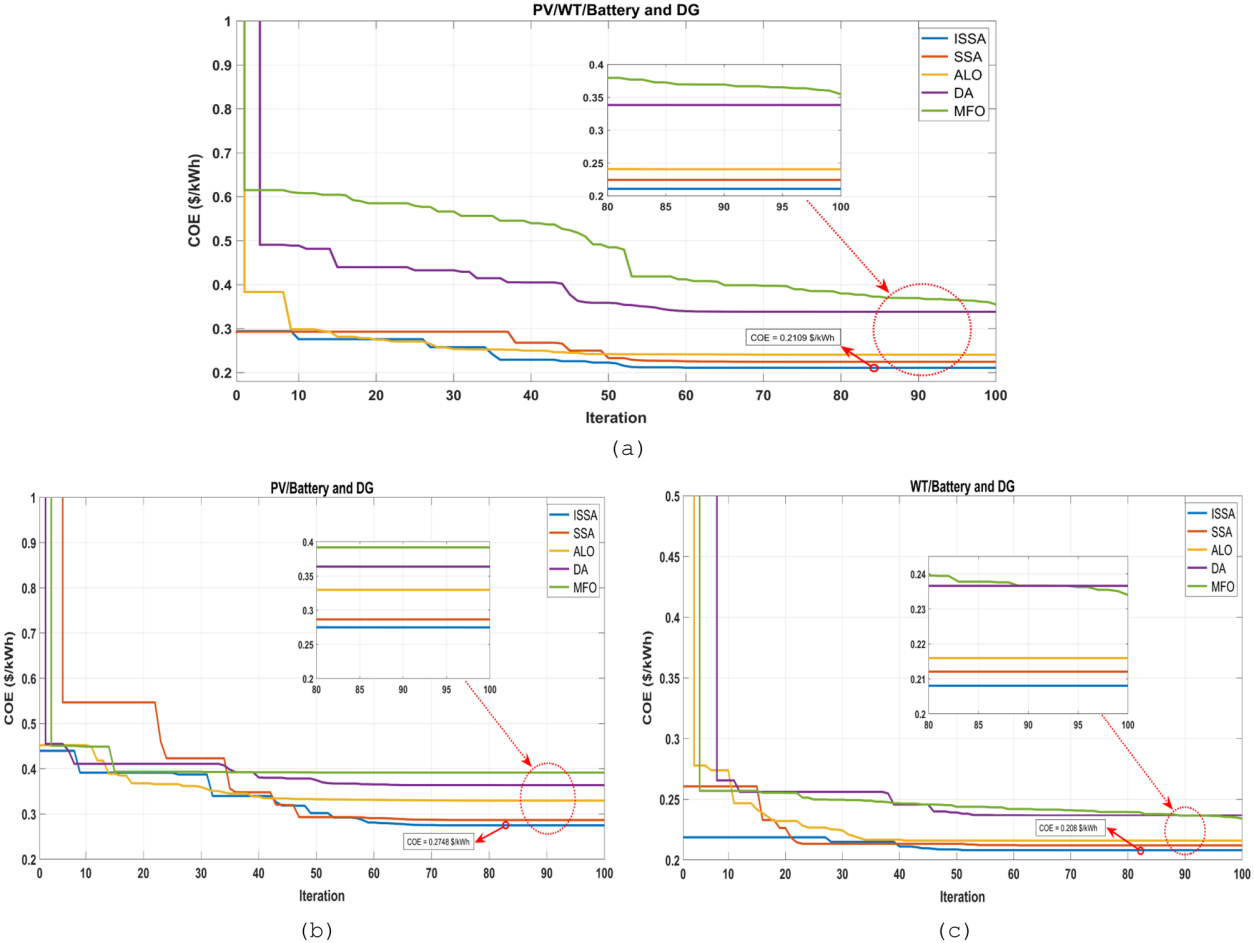
The objective function convergence using the proposed algorithms for all configurations, (**a**) PV/WT/BESU/DG, (**b**) PV/BESU/DG, (**c**) WT/BESU/DG.

To verify the effectiveness and ability of the ISSA algorithm to achieve the best optimum design with high reliability and minimal investment expenses. This is done by comparing its results with those obtained from the SSA, ALO, DA, and MFO algorithms for all the studied configurations. The convergence of the five optimization algorithms was analyzed using the convergence curves presented in Fig. 12. The convergence curves for the PV/WT/BESU/DG configuration are displayed in Fig. 12a. The ISSA algorithm consistently provides better convergence results. In this configuration, the SSA algorithm achieves the second-best convergence, while the convergence values for ISSA, SSA, ALO, DA, and MFO are 0.2109 $/kWh, 0.2245 $/kWh, 0.2407 $/kWh, 0.3385 $/kWh, and 0.3549 $/kWh, respectively. Figure 12b presents the convergence curves for the PV/BESU/DG configuration. The ISSA algorithm consistently converges to the best optimal solution within 60 iterations. The convergence values for ISSA, SSA, ALO, DA, and MFO are 0.2748 $/kWh, 0.2865 $/kWh, 0.3297 $/kWh, 0.3637 $/kWh, and 0.3917 $/kWh, respectively. The convergence curves for the WT/BESU/DG configuration are shown in Fig. 12c, where the ISSA algorithm consistently obtains the best solution. Convergence is achieved after 50 iterations, with convergence values of 0.2080 $/kWh for ISSA, 0.2121 $/kWh for SSA, 0.2160 $/kWh for ALO, 0.2366 $/kWh for DA, and 0.2340 $/kWh for MFO. The ISSA algorithm has been shown to be highly efficient and effective at providing better convergence results than other algorithms. It can be used for optimizing and providing optimal solutions for different microgrid configurations.

Table 6 summarizes the comparison results of five algorithms: ISSA, SSA, ALO, DA, and MFO, in different microgrid configurations. The results indicate that the most suitable configuration for the case study is PV/WT/BESU/DG using the ISSA algorithm. The COE of the system in this configuration is 0.2109 $/kWh, which is equivalent to 376,063.8 $ for the total NPC. The constraints have been met: LPSP is 4%, the system gets 96.0655% of its power from RS, and the BESU autonomy day is set at 1.5291. In the other configurations, the PV/BESU/DG receives more attention than the WT/BESU/DG, indicating the efficacy of the synergy between the PV and wind systems.

**Table 6:** Economical and technical results from the proposed algorithms for all configurations.

MS	Algorithm	COE ($/kWh)	NPC ($)	LPSP	REF (%)	AD (Day)
PV/WT/BESU/DG	ISSA	0.2109	376,063.8	0.04	96.0655	1.5291
	SSA	0.2245	400,358.9	0.0382	96.3846	1.5065
	ALO	0.2407	429,279	0.04	96.0002	1.5181
	DA	0.3385	603,819.7	0.04	97.8808	1.4673
	MFO	0.3549	632,910.7	0.039	96.5101	1.5006
PV/BESU/DG	ISSA	0.2748	490,099	0.06	95	1.5291
	SSA	0.2865	510,978.2	0.0583	95	1.5291
	ALO	0.3297	587,986.8	0.06	96.0075	1.5169
	DA	0.3637	648,693.7	0.0479	95	1.5291
	MFO	0.3917	698,656.4	0.0524	94.9988	1.4488
WT/BESU/DG	ISSA	0.2080	371,038.8	0.0388	90	1.3928
	SSA	0.2121	378,252.6	0.0486	90	1.273
	ALO	0.2160	385,178.6	0.0474	90.0004	1.2718
	DA	0.2366	421,958.7	0.05	89.9989	1.177
	MFO	0.2340	417,351.4	0.0498	91.1017	1.2078

Table 6 reveals that the COE ranges from 0.2080 $/kWh to 0.3917 $/kWh. The lowest COE is found in the WT/BESU/DG configuration at 0.2080 $/kWh, and the highest COE is found in the PV/BESU/DG configuration at 0.3917 $/kWh. The total NPC ranges from 698,656.4 $ to 371,038.8 $. The constraints are respected in all cases, with LPSP less than 6% in all cases. The two most favorable cases are 3.8% and 4% in the PV/WT/BESU/DG configuration using the ISSA and SSA algorithms, respectively. The aforementioned case is 6% in the PV/BESU/DG configuration using the ISSA algorithm. In all cases, the REF performed well, exceeding 89.9989%. According to the DA algorithm, this is the most efficient PV/BESU/DG configuration and the worst WT/BESU/DG configuration for REF performance. The BESU autonomy day results were all convergent. The table demonstrated that the PV/WT/BESU/DG configuration using the ISSA algorithm was the most optimal microgrid configuration, with the highest cost-effectiveness and reliability.

Table 7 provides a comparison summary with an emphasis on sizing outcomes using the same methods and configurations. ISSA algorithm finds the most suitable MS, composed of 10 PV systems, 9 WT, 24 BESU, and 3 DG. Additionally, this table displays the length of time required for each algorithm to complete the optimization process.

**Table 7:** Optimal sizing results from the proposed algorithms for the studied configurations.

MS	Algorithm	${N}_{pv}$	PV (kW)	${N}_{wt}$	WT (kW)	${N}_{batt}$	BESU (kW)	${N}_{DG}$	DG (kW)	Time (s)
PV/WT/BESU/DG	ISSA	10	73.0000	9	45.1400	24	49.9999	3	2.4221	950.5462
	SSA	10	73.0000	12	56.1855	23	49.2601	3	2.5213	955.0167
	ALO	14	96.5790	8	38.1487	23	49.6377	3	2.7940	1082.542
	DA	22	156.8879	12	56.0965	22	47.9773	3	2.6395	1212.283
	MFO	25	180.8246	5	25.2562	23	49.0679	4	3.7316	1319.526
PV/BESU/DG	ISSA	20	146.0460	–	–	24	49.9999	4	3.3284	1002.983
	SSA	21	152.6540	–	–	24	49.9980	4	3.4996	1055.774
	ALO	26	185.4879	–	–	23	49.6009	4	3.4523	1455.23
	DA	27	196.3262	–	–	24	49.9997	5	4.6178	1494.567
	MFO	30	212.9717	–	–	22	47.3736	5	5.0000	1667.075
WT/BESU/DG	ISSA	–	–	22	105.6795	21	45.5421	4	3.7800	1024.599
	SSA	–	–	22	108.6075	20	41.6247	4	3.9000	1076.804
	ALO	–	–	23	110.6090	20	41.5852	4	4.0000	1457.172
	DA	–	–	25	121.8705	18	38.4849	5	4.5418	1491.183
	MFO	–	–	25	124.9215	19	39.4913	5	4.1848	1623.207

For the PV/WT/BESU/DG configuration, the MFO algorithm generated the largest PV systems, 25, while the ISSA and SSA algorithms created the fewer PV systems, 10. The MFO method resulted in the lowest WT, 5, whereas the SSA approach achieved 12. All algorithms yielded convergent BESU. MFO produced 4 DGs, whereas ISSA produced 3.

For the PV/BESU/DG configuration, the MFO algorithm yielded the largest PV systems at 30, while the ISSA algorithm produced the fewest at 20. All algorithms resulted in convergent BESU. MFO produced 5 DGs, while ISSA had 4.

For the WT/BESU/DG configuration, the MFO algorithm gave the highest WT, 25. In contrast, the ISSA algorithm gave the lowest WT, 22. The ISSA algorithm produced the largest BESU, 21, while the MFO algorithm yielded the fewest, 19 BESU. MFO produced 5 DGs, while ISSA had 4.

The optimization time varied between the algorithms, with ISSA being the fastest, followed by SSA, and followed by other algorithms in all configurations. These results demonstrate the effectiveness of the ISSA algorithm at optimizing size.

### Comparative analysis of the contribution of microgrid energy generation

**Figure 13:**
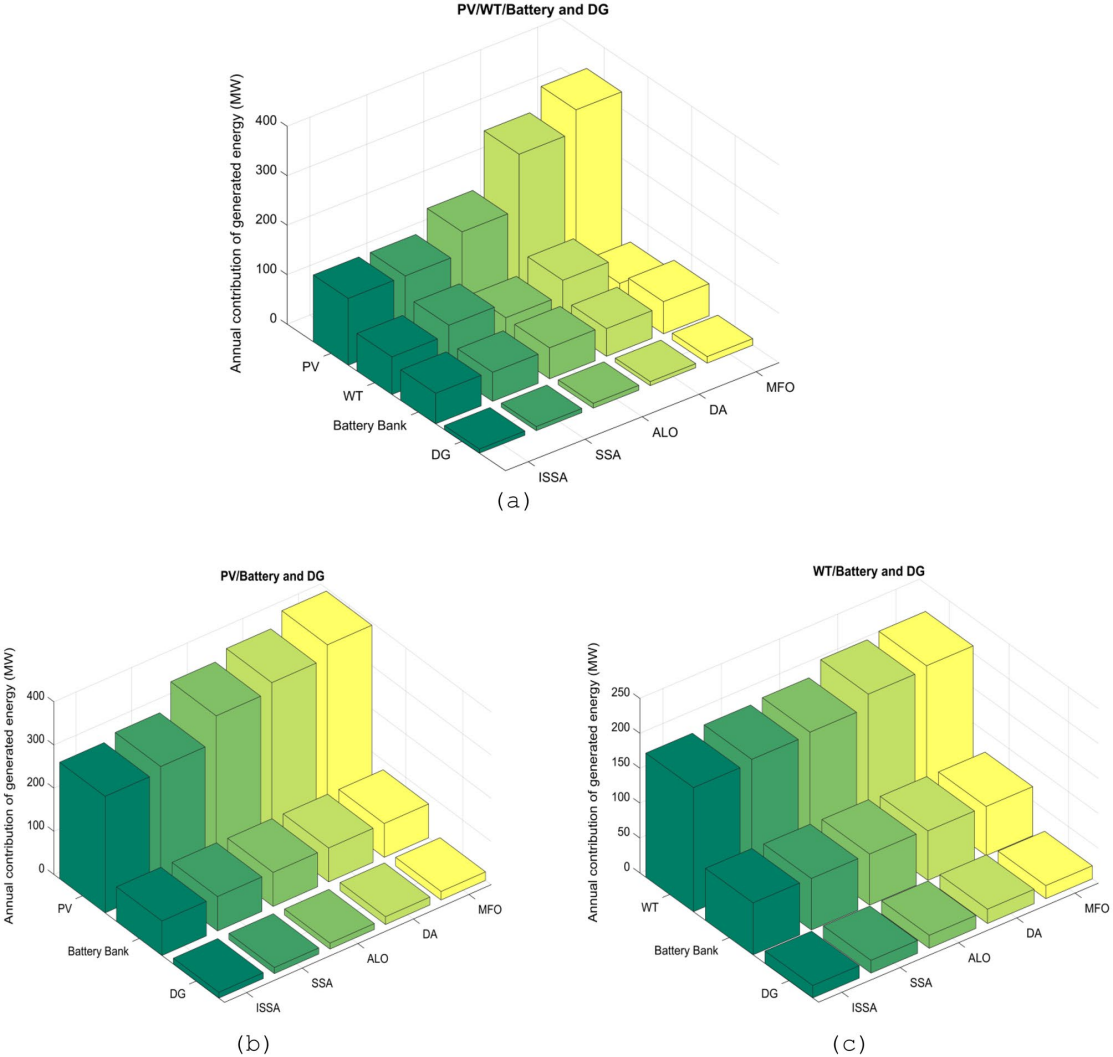
The annual energy generation contribution in different architectural combinations of the MS, (**a**) PV/WT/BESU/DG, (**b**) PV/BESU/DG, (**c**) WT/BESU/DG.

A comparison of the energy contributions from microgrid components using different design configurations. We analyzed three architectural combinations: PV/WT/BESU/DG, PV/BESU/DG, and WT/BESU/DG. Each architecture had five algorithms analyzed. Figure 13 shows the energy contributions of microgrid components throughout one year.

PV/WT/BESU/DG design provides the greatest energy since it uses four components. The PV and WT provide renewable energy, while the BESU and DG supply backup power when other sources fall short. The results are: The ISSA algorithm contributed 134.8739 MW, 75.9201 MW, 61.72 MW, and 8.2937 MW to PV, WT, BESU, and DG, respectively, and the SSA algorithm contributed 134.8739 MW, 94.4973 MW, 58.718 MW, and 8.2927 MW to PV, WT, BESU, and DG, respectively. PV and WT systems generated the highest energy across all algorithms. Figure 13a's comparison graph shows each algorithm's energy contribution differences.

PV/BESU/DG design produces less energy than the PV/WT/BESU/DG architecture since it uses just three components. This architecture does not use WT. When the PV is not producing enough electricity, the BESU and DG supply backup. ISSA algorithm generated an annual contribution of energy of 269.8327 MW, 79.965 MW, and 13.4916 MW for PV, BESU, and DG, respectively; the results of all algorithms are illustrated in Fig. 13b.

WT/BESU/DG design produces the least energy since it uses WT, BESU, and DG to create power. This architecture does not use PV. The BESU and DG offer backup power when the WT is not producing enough energy, but the WT is still a renewable source. ISSA algorithm contributed 177.7404 MW, 74.4557 MW, and 17.774 MW to WT, BESU, and DG, respectively; Fig. 13c shows how the different microgrid elements contribute energy with all algorithms.

The proposed ISSA algorithm outperforms competing methods in the energy contribution efficiency of microgrid elements. The ISSA algorithm may be a potential approach for optimizing MS and enhancing their performance, essential for sustainable and dependable energy systems. PV/WT/BESU/DG configuration produces the most energy compared to PV/BESU/DG and WT/BESU/DG. Such signs suggest that the PV/WT/BESU/DG configuration can be an effective solution for MS, especially in areas with high renewable energy potential.

### Comparative analysis of the CO_2_ emissions, fuel consumption, and COE

**Figure 14:**
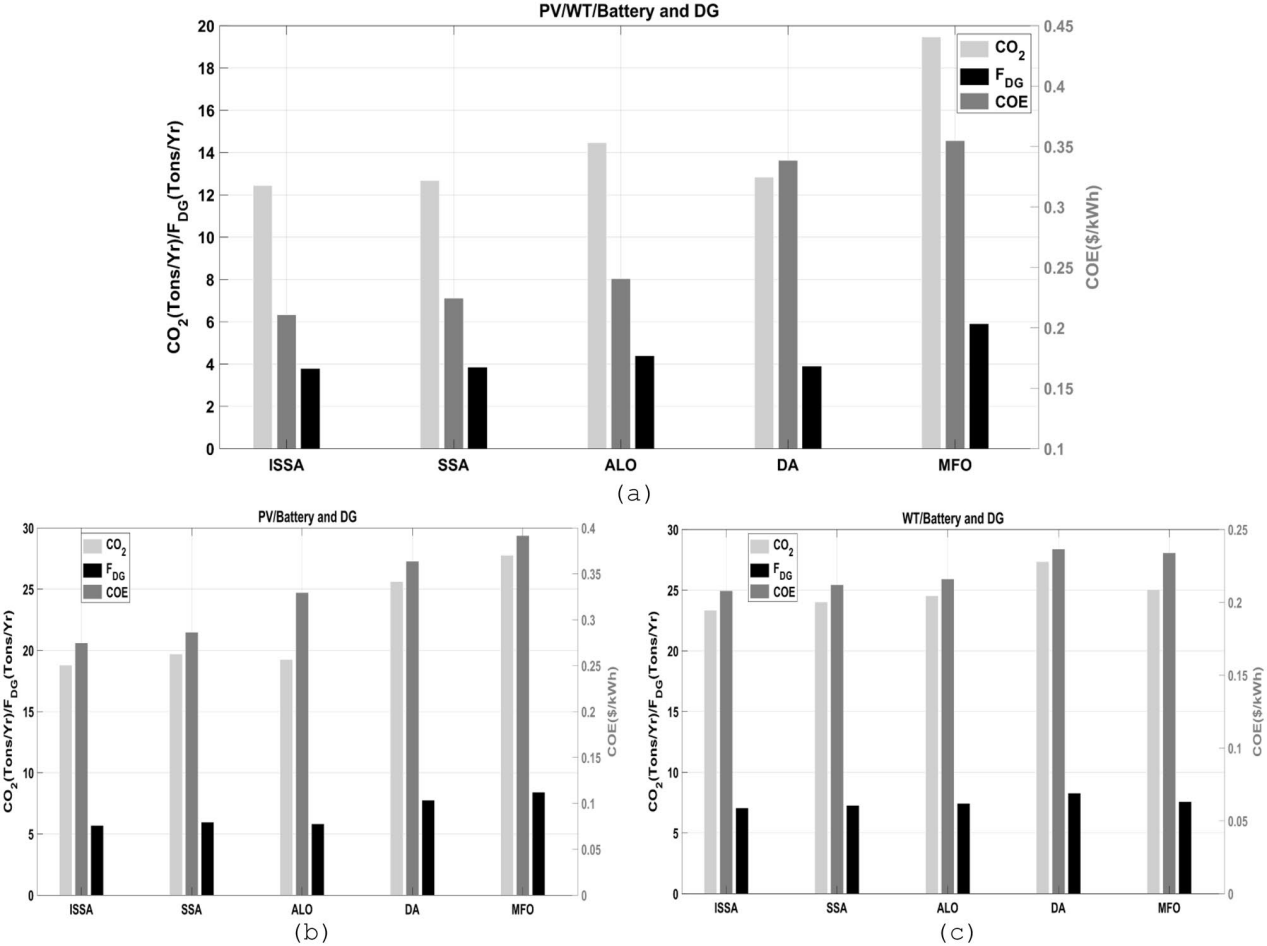
The comparative analysis of the five proposed algorithms in terms of annual CO_2_ emissions, fuel consumption, and COE in different MSs, (**a**) PV/WT/BESU/DG, (**b**) PV/BESU/DG, (**c**) WT/BESU/DG.

An analysis was performed to compare the CO_2_ emissions, fuel consumption, and COE of the microgrid using different architecture combinations: PV/WT/BESU/DG, PV/BESU/DG, and WT/BESU/DG. The five proposed algorithms for each architecture were analyzed to determine their respective performances. As shown in Fig. 14.

The first configuration of PV/WT/BESU/DG. This configuration has the lowest CO_2_ emissions and fuel consumption. The high cost of PV and WT components made this design the second-highest COE. Results of the ISSA algorithm of 12.4457 Tons/Year CO_2_ emissions, 3.7731 Tons/Year fuel consumption, and 0.21085 $/kWh COE. Figure 14a shows the comparative results with all algorithms.

The second configuration of PV/BESU/DG. The analysis demonstrated minimal CO_2_ and fuel usage for this configuration. This configuration earned the greatest COE since it used only solar energy. The outcomes of the ISSA algorithm are 18.8022 Tons/Year of CO_2_ emissions, 5.7002 Tons/Year of fuel consumption, and 0.27478 $/kWh COE. Figure 14b shows the comparative results with all algorithms.

The third configuration of WT/BESU/DG. The research found that this configuration's dependency on a few renewable resources increased CO_2_ emissions and fuel usage. Due to its wind-only design, this architecture has the lowest COE. The outcomes of the ISSA algorithm are 23.3429 Tons/Year of CO_2_ emissions, 7.0768 Tons/Year of fuel consumption, and 0.20803 $/kWh COE. Figure 14c shows the comparative results with all algorithms.

ISSA is more efficient and environmentally friendly than other algorithms in various MS configurations, according to the results. Today, since carbon footprints and RES are becoming more relevant, this is crucial. This research stressed the relevance of cost and environmental effects in MS design.

### Analysis of the total NPC

**Figure 15:**
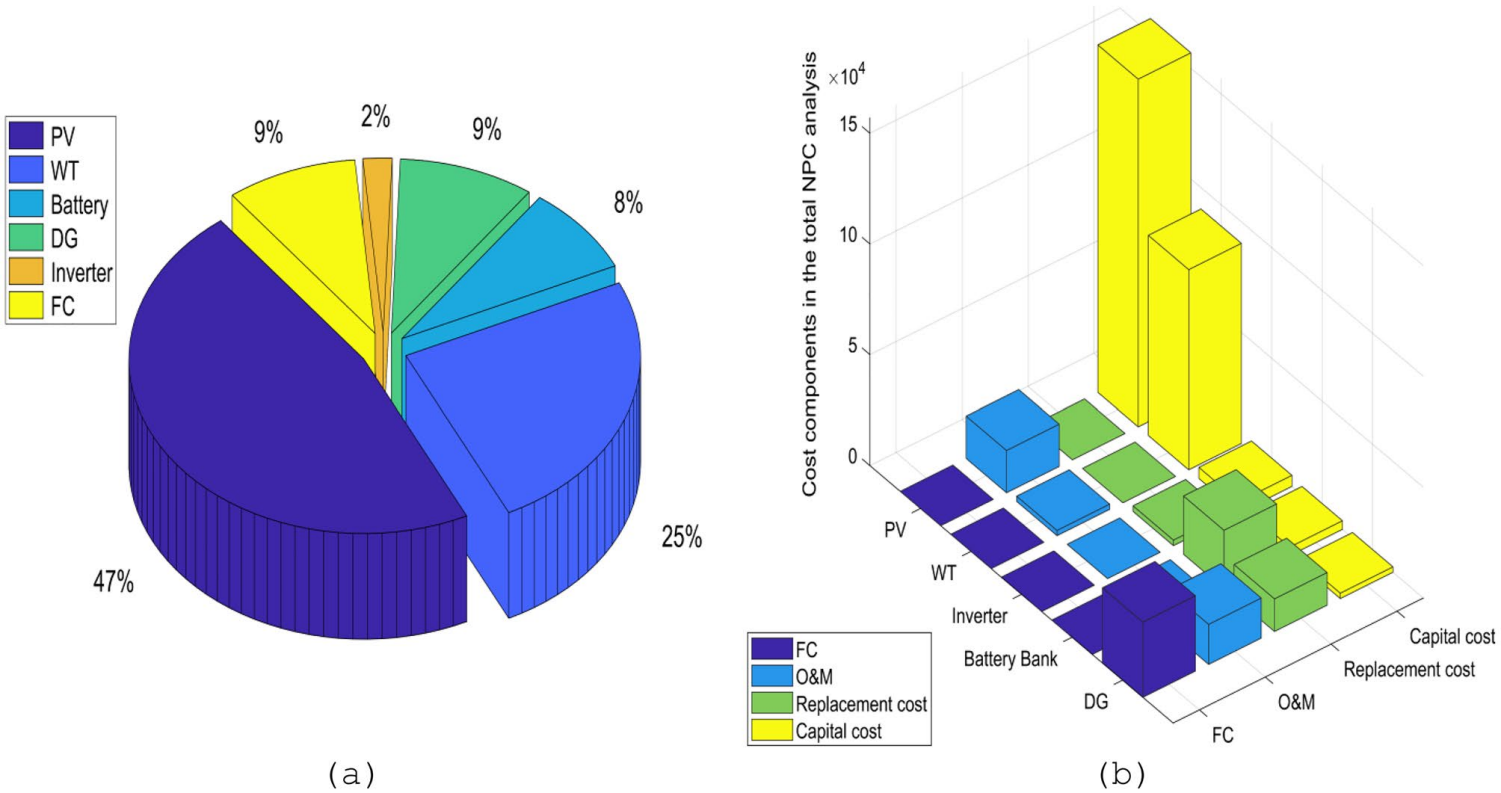
Analysis of the total NPC, (**a**) the contributions of the different elements of the microgrid to cash flow, (**b**) the cash flow associated with the various components of the microgrid.

In the following section, we will talk about the total NPC of the microgrid over the course of its lifetime. The analysis was done using the results from the ISSA, which were found to be better than the studied methods of sizing for microgrid optimization, which had a total NPC of 376,063.8 $. The total NPC of the MS is made up of four main components: the cost of capital, the cost of replacement, the cost of O&M, and the cost of fuel. Specifically, the capital costs, replacement costs, O&M costs, and fuel costs account for 69%, 11%, 11%, and 9% of the total NPC, respectively. Figure 15 presents a detailed analysis of the total NPC associated with the various components of the microgrid. Figure 15a illustrates the respective contributions of the PV array, WT, FC, DG, BESU, and Inverter to the total NPC, which are 47%, 25%, 9%, 9%, 8%, and 2%, respectively. The PV and WT, which serve as RES power supplies, occupy the largest proportion of the total NPC.

Figure 15b shows the cash flow division of the MS in the area of interest for the given case study. As shown in the diagram, all of the microgrid's parts, except for the inverter, have costs for O&M. Also, it should be noted that the BESU, DG, and Inverter are the only components that have replacement costs. The PV and DG incur a significant portion of the operations and maintenance expenses. Additionally, higher fuel consumption costs. The outcomes are as follows: for the capital costs, the costs are divided among different types of equipment: the cost of PV is 156,950 $, the cost of WT is 90,280 $, the cost of the inverter is 2500 $, the cost of the BESU is 3865.733 $, and the cost of DG is 2422.06 $. These costs are for the initial investment required to purchase the equipment. The replacement costs for the equipment are given as 2500 $ for the inverter, 26,321.1 $ for the BESU, and 14,733.32 $ for the DG. These are the costs involved in replacing the equipment after a certain period of time or when the equipment has reached the end of its useful life. O&M costs are 19,080.06 $ for PV, 2359.655 $ for WT, 505.1947 $ for the BESU, and 18,072.47 $ for DG. These costs represent the ongoing costs associated with operating and maintaining the equipment. The fuel costs associated with the DG are 33,974.12 $ worth of fuel. This is for the cost of the diesel fuel that is required to run the generator.

This shows that the developed MS is a good option and can compete with the systems that are used in most microgrid communities right now. So, the project we just talked about is thought to be economically possible in the proposed case study area. If the microgrid project is put into place correctly, it should make the energy supply more efficient and help reach the sustainable development goals.

### Analysis of power flow

**Figure 16:**
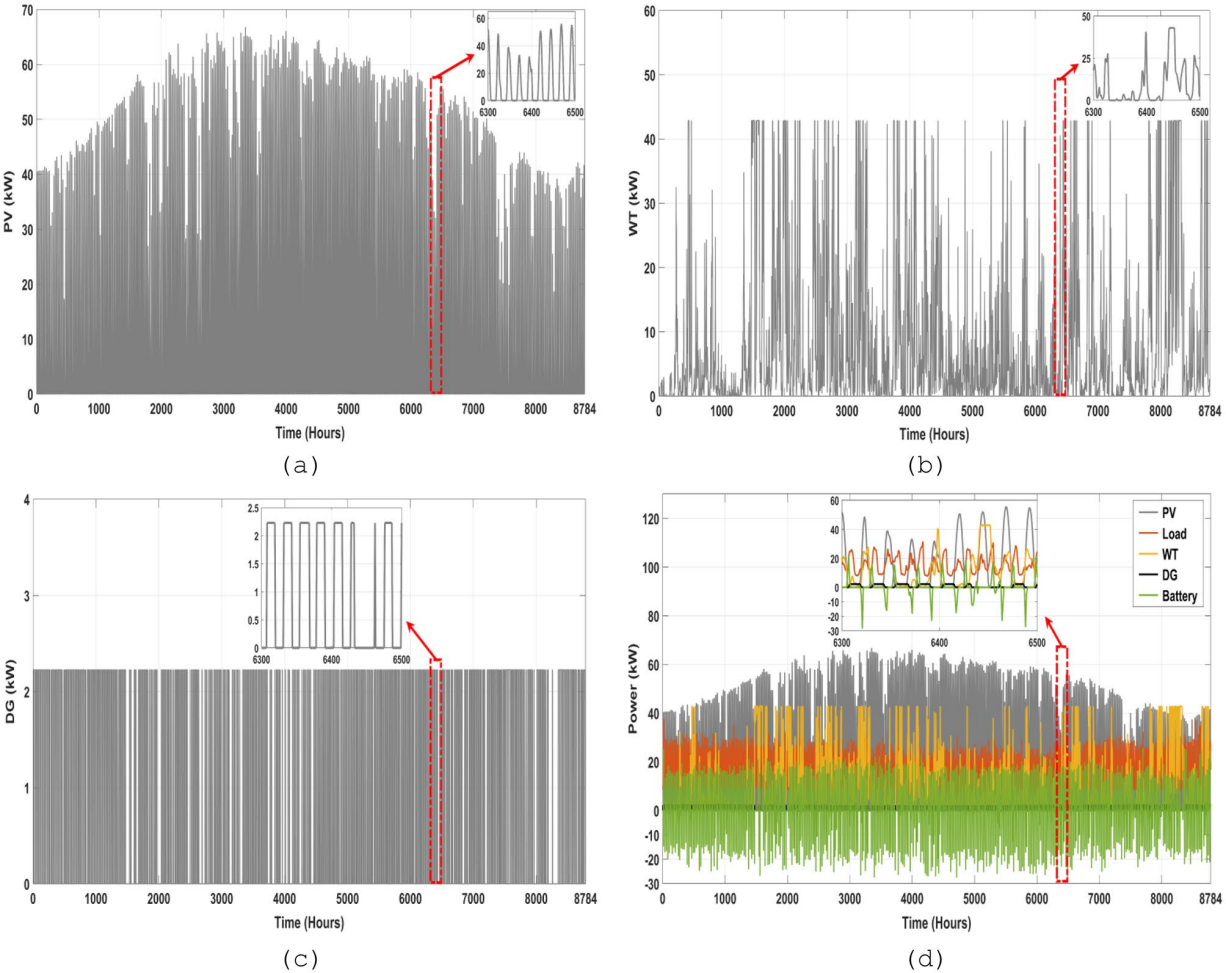
The power generation by different components of the MS over the course of a year, (**a**) the PV, (**b**) the WT, (**c**) the DG, and (**d**) the annual electricity generation mix.

This part focuses on the evaluation of the energy flow performance of the optimized MS according to the optimal combination of microgrid components. The optimal configuration was determined using the ISSA algorithm that was investigated in the section above; this analysis is based on ISSA algorithm results for one year, which were found to be the most suitable among the various optimization algorithms that were tested. Figure 16a,b depict the PV and WT's annual power production according to the best system size. Figure 16c depicts the backup system's (DG) power contribution, while Fig. 16d depicts the annual mix of power produced from PV, WT, BESU, and DG, as well as the load power demand. This analysis may be expanded throughout the course of the project's lifetime, and it will need to consider the sizes of the microgrid components as established by the proposed ISSA method. This will aid in avoiding supply and demand imbalances within the microgrid.

**Figure 17:**
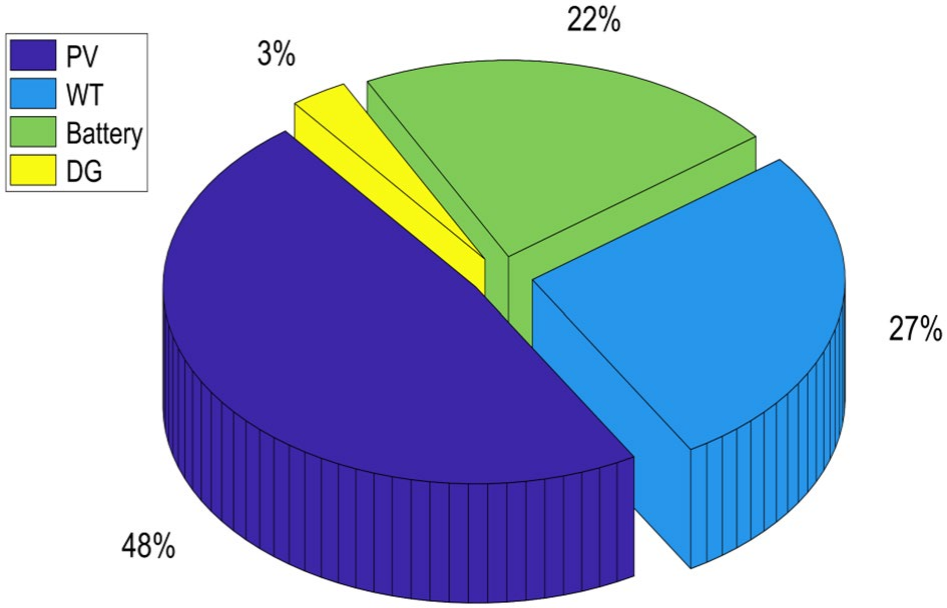
The total annual power generation contribution of the PV, WT, BESU, and DG.

Figure shown in Fig. 17 gives a clear representation of the contributions made by various sources to the microgrid's overall power generation. The data indicates that the PV contributes 48% of the microgrid's total energy production, which is a significant contribution. The WT, BESU, and DG are other elements of power generation. The WT accounts for around 27% of the total energy generated, while the BESU and DG contribute 22% and 3%, respectively. The PV contributes significantly to the total energy generated, with an annual energy output of 134.8739 MW/Year. The WT, BESU, and DG also contribute a considerable amount of energy, with 75.9201 MW/Year, 61.7200 MW/Year, and 8.2937 MW/Year, respectively. It is evident that the PV array is the primary source of energy generation, while the WT, BESU, and DG also play a crucial role in ensuring a stable and consistent supply of energy.

**Figure 18:**
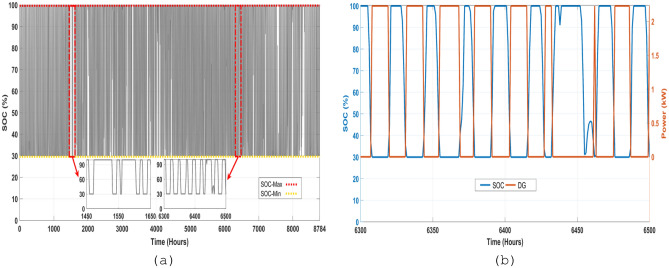
The hourly variation of the battery SOC and generated power by DG, (**a**) the annual profile of the battery SOC, (**b**) the contribution of the DG and battery SOC in the same period.

BESU is crucial to the HRES because it stores any surplus energy produced by the system. The battery's SOC is shown in Fig. 18a. Depending on the load needed and the electricity provided by the PV and WT, the analysis reveals that the SOC of the BESU might vary significantly. The BESU is charged during times of surplus electricity production and drained during times of peak demand. This makes sure that the BESU can kick in when the energy output from the PV and WT isn't enough to fulfill the needs of the intended region.

Figure 18a displays the battery SOC data for a full year. Hours make up the x-axis of the graph, while BESU state-of-charge percentages make up the y-axis. The selected time range of [1450–1650] allows us to examine the SOC of the BESU in more detail, as seen in the same picture at a larger scale. The figure depicts a little variation in SOC of the BESU between about 30% and 100% throughout this time and more so between the time intervals [6300–6500], with some times exhibiting a fall in SOC and others displaying an increase.

Figure 18b presents an analysis of the battery SOC and the contribution of the DG power for the same period. This figure provides insight into how the battery bank's SOC and the DG work together to fulfill load energy demand in the system. The DG is used as a backup system to provide power during periods of low power generation or high energy demand, such as at night or during periods of low wind or solar irradiation. The figure shows that the battery bank SOC and the DG work together tofulfill the energy demand. For example, during periods of high energy demand, the DG provides additional power to the system, while the SOC of the BESU decreases as it is discharged to meet the demand. On the other hand, when there is little demand for energy, the SOC of the BESU rises as a result of the excess power that the PV and WT produce, and the DG may not even be necessary. But it's important to keep in mind that the SOC of the BESU depends on many things, like how much energy the target area needs, how much power the HRES makes, and how much power the BESU can hold.

**Figure 19:**
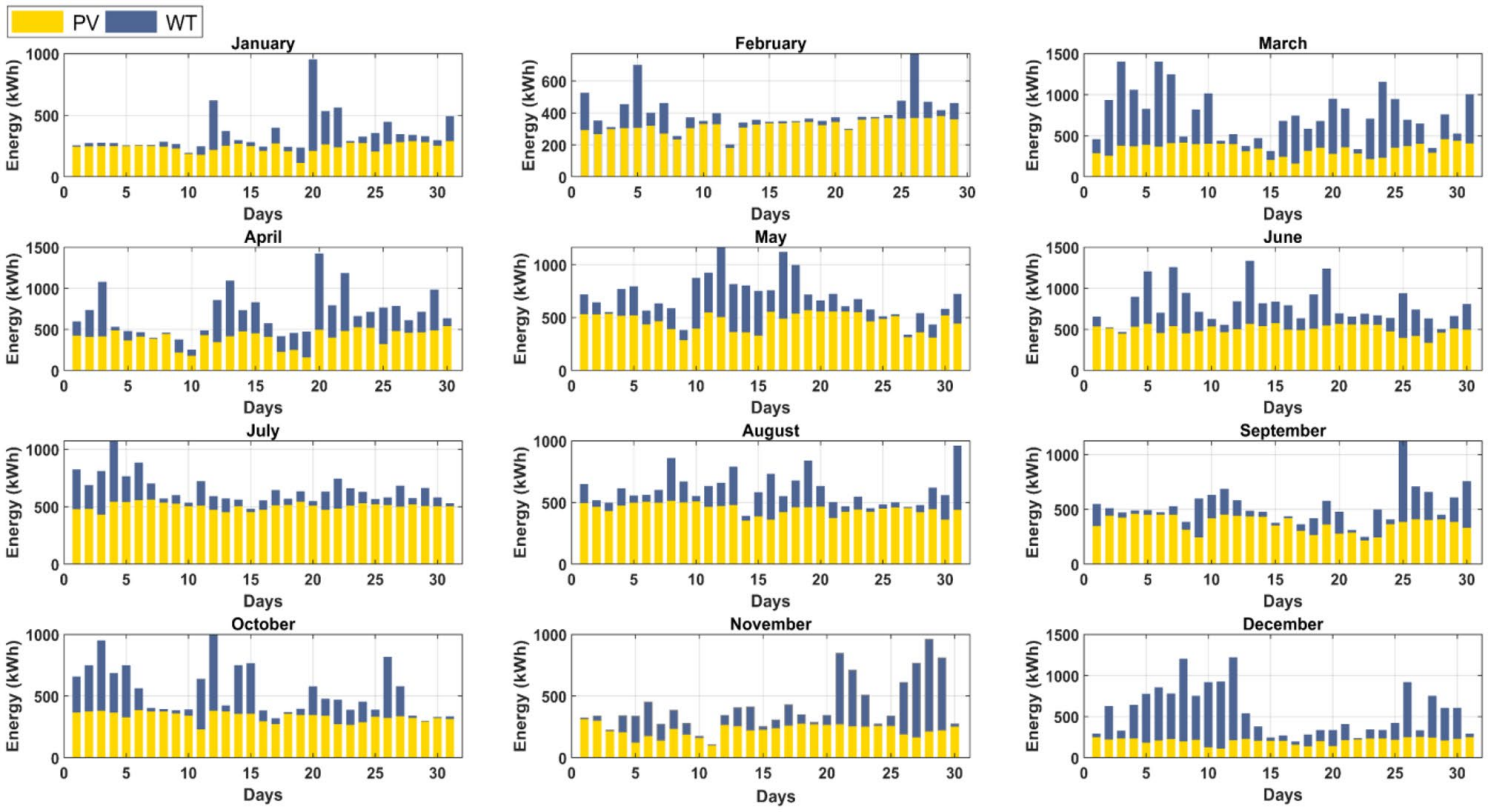
Daily electricity output from wind and solar between January and December.

On the other hand, monthly presentations of PV power production and WT are given from January through December and are illustrated in Fig. 19. According to the research, the biggest monthly total PV power output occurs in July, suggesting that PV power generation is greater during the hot season than during the cold season. The PV system produced close to 15.7889 MWh/month throughout this month. On July 7th, solar electricity output peaks at 566.285 kWh/day, the highest level ever recorded. In contrast, electricity generation from PV is at an all-time low in December. This month, PV systems produced about 6.6308 MWh/month. On December 11th, the lowest PV generation rate of the month was 118.0584 kWh/day.

The study found that March has the highest monthly total WT power production, indicating that WT power generation is higher in the cold season than in the hot season. This month, the WT system generated around 12.5407 MWh/month. The greatest ever recorded production of wind-generated power was 1.0267 MWh/day on March 6. However, in February, WT's power production hit a record low. This month, WT systems have a production of around 2.162 MWh/month. The lowest daily WT generating rate this month is 1.4399 kWh/day on February 17th. The combination of the highest total power generated from PV sources and WT was about 23.4675 MWh/month in June. 10.7114 MWh/month is the bare minimum power this combination can produce in January.

These results provide valuable insights into the power generation trends of PV and WT systems. The integration between PV and wind energy during the cold and hot seasons suggests that the system is capable of generating a consistent amount of power throughout the year. However, there is variation in some months, highlighting the significance of choosing the optimal size for the system to guarantee it can meet the necessary energy demand.

## Conclusion

This study offers the ISSA, a metaheuristic optimization technique inspired by nature, to determine the optimal size for an independent microgrid in Djelfa, Algeria. Three configurations of the MS have been implemented, such as: (1) PV/WT/BESU/DG, (2) PV/BESU/DG, and (3) WT/BESU/DG, which consist of PV, WT, BESU, and DG. The optimization problem's primary focus was meeting the energy demand in an off-grid community. The suggested approach determines four design variables: the number of PV, WT, BESU, and DG. As a result, reliability and renewable fraction are maximized while COE is minimized. The obtained results show that the PV/WT/BESU /DG configuration is the most cost-effective and reliable system, with a COE of 0.2109 $/kWh, an NPC of 376,063.8 $, a LPSP of 4%, a REF of 96.0655, and an AD of 1.5291 days. Otherwise, the best MS configuration contains 10 PV, 9 WT, 24 BESU, and 3 DG. These systems work together to provide sustainable energy sources. This system consumes only 3.7731 Tons/Year of fuel, offset by an impressive 12.4457 Tons/Year of CO_2_ emission reduction. Additionally, the results showed that in all configurations studied, the ISSA algorithm reached the optimal solution faster and more efficiently than the traditional SSA, ALO, DA, and MFO algorithms. Furthermore, analyses of CO_2_ emissions, fuel consumption, COE, total NPC, and energy flow of the proposed system configurations were performed to obtain their impacts on the MS performance, which were confirmed to be particularly important and have a significant impact on the overall system performance. This suggests that the proposed microgrid is highly efficient and environmentally friendly. This makes it an ideal solution for remote areas or places with unreliable grid power supplies. Overall, this MS offers an effective solution to address energy challenges while promoting sustainability and reducing carbon footprints in the long run. This study will provide useful information for decision-makers working to develop Algeria's renewable energy sector. In future studies, the proposed ISSA may be applied to other engineering problems.

## Perspectives and future work

HRES helps reduce fossil fuel use, essential to a sustainable future. Future research should include varied energy sources. Matches expectations in each area. Scientists, politicians, and business leaders must collaborate to discover and deploy the most promising sources. Promote renewable energy and educate the public about its benefits. Optimization of HRES' design and operation using AI algorithms and hybrid optimization methods may boost efficiency and reduce costs. AI can forecast energy demand and optimize RES. Hybrid optimization can balance cost, reliability, and environmental effects. These strategies help us control hybrid system complexity. We customize energy solutions for each place. With this, we can accelerate the transition to a sustainable energy system and improve future generations' lives.

**Figure 20:**
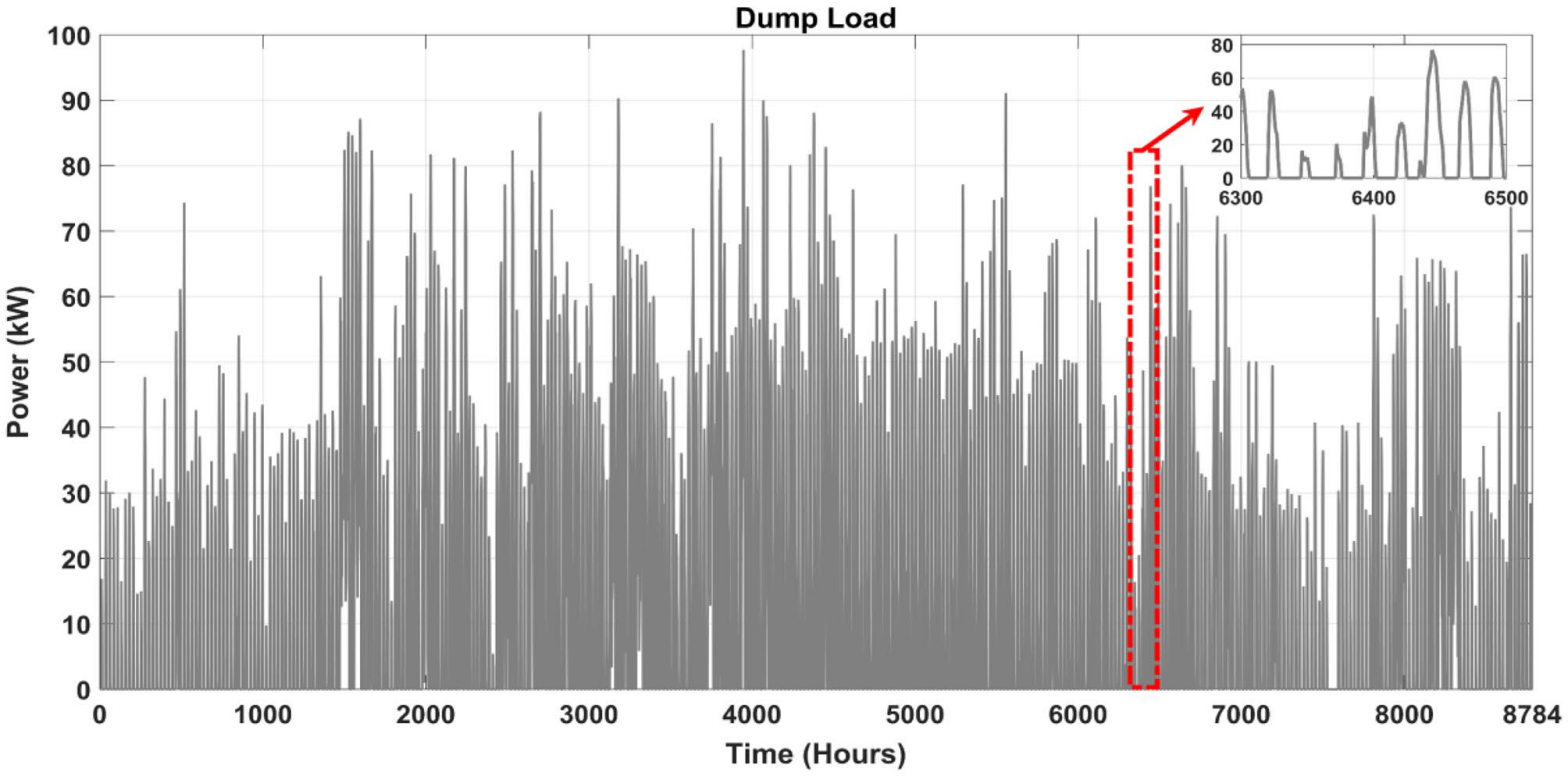
The annual excess energy used as dump load by the ISSA algorithm for PV/WT/BESU/DG configuration.

However, surplus energy is a significant concern with MS. Low demand or excessive energy output often generates this. Wasted or discarded energy increases MS expenses and resource usage inefficiency. Our technology heats buildings and powers irrigation using this energy. Unfortunately, past research has ignored surplus energy in MS. Figure 20 shows the yearly energy drain. The ISSA algorithm wastes and produces surplus energy in an MS over a year. The average energy dump for the year is 13.209 kW, and the total is 116.02 MW.

Propose linking rural microgrids in remote areas, where communities depend on localized energy sources like solar panels and WT. This network of interconnected systems enables communication between neighboring farms and facilitates energy sharing. In times of energy shortage, a microgrid can tap into a nearby microgrid's surplus energy to meet demand, promoting a comprehensive EMS. This approach guarantees a continuous, reliable electricity supply for isolated communities.

### Supplementary Information

Supplementary Information.

## Data Availability

The wind speed, solar radiation, and ambient temperature data used in this work are available on the following website: http://www.soda-pro.com/web-services/meteodata, accessed on April 5, 2023. The additional data offered in this research may be found in the main content of this publication.
