# Supplementary material for: Techno-economic optimization for isolated hybrid PV/wind/battery/diesel generator microgrid using improved salp swarm algorithm
Source: Sci Rep. 2024 Feb 5;14:2920. doi: 10.1038/s41598-024-52232-y (PMC10844505; doi:10.1038/s41598-024-52232-y)
Supplement: Supplementary file 1 — Supplementary Information. [file 41598_2024_52232_MOESM1_ESM.docx]

# **Appendix A. Component Specifications for Microgrid System**

**Table.S1** Technical and economic specifications of MS components.

| **Component** | **Parameters** | **Values** | **Unit** |
| --- | --- | --- | --- |
| PV | Rated power at STC | 7.3 | kW |
|  | Initial cost | 2150 | $/kW |
|  | Lifetime | 25 |  |
|  | Temperature coefficient | $-3.7\times{10}^{-3}$ | 1/°C |
|  | Regulator cost | 1500 | $ |
|  | Yearly maintenance cost per kW  Efficiency | 20  95 | $/Year  % |
| WT | Rated power | 5 | kW |
|  | Rated wind speed | 9.5 | m/s |
|  | Cut-in wind speed | 2.5 | m/s |
|  | Cut-out wind speed  Initial cost | 40  2000 | m/s  $/kW |
|  | Blades diameter | 6.4 | m |
|  | Swept area | 12.6 | m^2^ |
|  | Lifetime  Tower height | 25  20 | Year  m |
|  | Regulator cost | 1000 | $ |
|  | Annual maintenance cost  Efficiency | 20  95 | $/Year  % |
| BESU | Nominal capacity | 360 | Ah |
|  | Nominal voltage | 6 | V |
|  | System voltage | 48 | V |
|  | Capital cost | 167 | $ |
|  | Replacement cost | 67 | $ |
|  | Annual maintenance cost | 1.67 | $/Year |
|  | Maximum state of charge | 100 | % |
|  | Minimum state of charge  Maximum DOD | 30  70 | %  % |
|  | Hourly self-discharge rate ($\sigma$)  Lifetime  Efficiency | 0.007  2  85 | %/Hour  Year  % |
| DG | Rated power | 4 | kW |
|  | Initial cost | 1000 | $/kWh |
|  | Lifetime  Replacement cost | 24000  1000 | Hours (5 year)  $/kWh |
|  | Maintenance cost | 0.065 | $/h |
|  | Fuel cost | 0.689 | $/L |
| Inverter | Initial cost | 2500 | $ |
|  | Efficiency | 92 | % |
|  | Lifetime | 12.5 | Year |
| Economic Parameters | Nominal interest rate | 10 | % |
|  | Annual inflation rate | 4 | % |
|  | Project lifetime | 25 | Year |
